# The effects of the prognostic biomarker SAAL1 on cancer growth and its association with the immune microenvironment in lung adenocarcinoma

**DOI:** 10.1186/s12885-023-10741-5

**Published:** 2023-03-27

**Authors:** Song Tong, Ni Jiang, Jun-Hao Wan, Chong-Rui Chen, Si-Hua Wang, Chuang-Yan Wu, Qiang Guo, Xiao-Yue Xiao, Huan Huang, Ting Zhou

**Affiliations:** 1 Department of Thoracic Surgery, Union Hospital, Tongji Medical College, Huazhong University of Science and Technology, Wuhan 430022, China; 2 Department of Obstetrics and Gynecology, Women and Children’s Hospitalof, Chongqing Medical University, Chongqing 401147, China; 3 Department of Thoracic Surgery, People’s Hospital of Dongxihu District, Wuhan 430040, China; 4 Department of Critical Care Medicine, Union Hospital, Tongji Medical College, Huazhong University of Science and Technology, Wuhan 430022, China

**Keywords:** SAAL1, Lung adenocarcinoma, Immune infiltration, Prognosis, Cell cycle

## Abstract

**Background:**

Inhibition of Serum Amyloid A-like 1 (SAAL1) expression could inhibit cancer progression and improve the prognosis of cancer patients. At present, the correlation between SAAL1 and lung adenocarcinoma (LAC) remains unclear. Therefore, this study surveyed the worth and pathway of SAAL1 in LAC progression and immunity.

**Methods:**

Bioinformatics and immunohistochemistry were used to identify the SAAL1 expression in LAC. The roles of SAAL1 expression in the existence values of LAC patients were explored, and the nomograms were constructed. Clinical values of SAAL1 co-expressed genes were evaluated by COX regression, survival, and Receiver operating characteristic (ROC) analysis. EDU and western blotting methods were used to inquiry the functions and pathways of the SAAL1 in cell growths. The correlation between the SAAL1 level and immune microenvironment was visualized using correlation research.

**Results:**

SAAL1 level was elevated in LAC tissues, and was observed in cancer tissues of dead patients. SAAL1 overexpression had something to do with shorter overall survival, progression-free interval, and disease-specific survival in LAC. The area under the curve of SAAL1 was 0.902 in normal tissues and cancer tissues. Inhibition of SAAL1 expression could inhibit cancer cell proliferation, which may be related to the decreased expression of cyclin D1 and Bcl-2 proteins. In LAC, SAAL1 level had something to do with stromal, immune, and estimate scores, and correlated with macrophages, T cells, Th2 cells, CD8 T cells, NK CD56dim cells, DC, eosinophils, NK CD56bright cells, pDC, iDC, cytotoxic cells, Tgd, aDC cells, B cells, Tcm, and TFH levels. SAAL1 overexpression had something to do with existence values and the immunity in LAC.

**Conclusions:**

Inhibition of SAAL1 expression could regulate cancer growth via cyclin D1 and Bcl-2. SAAL1 is a promising prognostic biomarker in LAC patients.

**Supplementary Information:**

The online version contains supplementary material available at 10.1186/s12885-023-10741-5.

## Introduction

In recent years, people have paid closer attention to their health. Chest computed tomography (CT) may reveal the presence of ground-glass nodules (GGNs) or mixed GGNs in the lungs. In most cases, the postoperative pathological evaluation of these GGNs demonstrates adenocarcinoma in situ, microinvasive, and invasive lung adenocarcinoma (LAC). In addition, gene detection of epidermal growth factor receptor (EGFR), tumor protein p53 (TP53), KRAS, and other gene mutations could predict the prognosis of LAC patients [1, 2]. These targeted therapies could enhance the existence values of LAC patients. However, the prognosis of patients with advanced LAC remained suboptimal. Therefore, new therapeutic targets are required to improve the prognosis of LAC patients.

Some genes could predict poor prognosis in LAC patients and are related to the occurrence and development of LAC [3–6]. SAAL1 was recently reported to be an inflammatory gene related to disease progression [7–10]. For example, Chen et al. reported that the level of SAAL1 in lung tissue of mice stimulated by lipopolysaccharide (LPS) increased. Inhibition of SAAL1 expression could reduce lung injury, edema and neutrophil infiltration, and inhibit LPS-stimulated inflammatory factor production and NLR signaling pathway [10]. SAAL1 expression was significantly increased in hepatocellular carcinoma (HCC) and had something to do with shorter overall survival (OS) in HCC. Interference with SAAL1 expression reduced the proliferation, migration, colony formation, and invasion ability of HCC cells. It also induced hepatocyte growth factor (HGF)/MET to drive AKT/mechanistic target of rapamycin kinase (mTOR) signaling pathway and heighten the sensitivity of HCC cells to sorafenib [8]. At present, the mechanisms of SAAL1 in LAC have not been reported. Therefore, bioinformatics was applied to investigate the SAAL1 levels in cancer tissues. The roles and signaling mechanisms of SAAL1 expression in the prognosis and cell growth in LAC were revealed. The correlation between the SAAL1 level and immune microenvironment was revealed to provide a new candidate marker for the treatment of LAC patients.

## Methods

### The expression levels of SAAL1

The gene expression data of all patients were gained from the Cancer Genome Atlas (TCGA), XENA-TCGA, and Genotypic Tissue Expression (GTEx) databases. The transcripts per million (TPM) and fragments per kilobase of transcript per million fragments mapped (FPKM) data types of cancer patients were included in this research. The TCGA database contained 730 normal and 10,363 tumor tissue samples. The XENA-TCGA database contained 727 normal and 9807 tumor tissue samples. The GTEx database contained 7568 normal tissue samples. SAAL1 levels in cancer tissues and normal tissues of paired and unpaired patients were analyzed via the Wilcoxon rank sum test and displayed by using the violin diagrams. In addition, the expression levels in the cancer tissues and normal tissues of 14 paired LAC patients undergoing surgical resection of our hospital via immunohistochemical.

### The clinical values of SAAL1 in LAC

Receiver operating characteristic (ROC) analysis was a tool for evaluating gene expression in cancer [11, 12]. In 59 normal and 535 LAC tissues from the TCGA database, ROC analysis were applied to survey the diagnostic value of SAAL1 expression in LAC. After combining the data of SAAL1 expression and clinicopathological characteristics of LAC patients from the TCGA database, Kaplan–Meier (K-M) survival analysis was applied to survey the prognosis values of SAAL1 expression in LAC, and The relationship between high- and low- SAAL1 levels and the T stage, N stage, M stage, pathologic stage, primary therapy outcome, gender, race, age, anatomic neoplasm subdivision, smoker, OS event, DSS event, and PFI event of LAC patients was analyzed. The grouping of cancer patients was based on the median value of SAAL1 expression. In addition, Univariate COX regression analysis was performed to evaluate the relationship between T stage, N stage, M stage, age, gender, and SAAL1 expression levels with the prognosis of LAC patients, and multivariate COX regression analysis was performed using *P* < 0.05 as the filtering criterion.

### The correlation between the SAAL1 level and existence values in patient subgroups

In 535 LAC patients, the correlation between SAAL1 overexpression and poor OS, progression-free interval (PFI), and disease-specific survival (DSS) was investigated in LAC patient subgroups based on the clinicopathological features (such as the T1, T2, T3, and others).

### The roles, pathways and network of SAAL1 co-expressed genes

The filter criteria for SAAL1 co-expressed genes was an absolute correlation coefficient greater than 0.5 using correlation analysis, which were defined as strongly co-expressed genes [13]. Gene Ontology, and Kyoto Encyclopedia of Genes and Genomes methods were performed to elucidate the roles and pathways of the strongly correlated genes of SAAL1 [13]. The protein network of the strongly co-expressed genes of SAAL1 was established in the Search Tool for the Retrieval of Interacting Genes (STRING) database.

### The roles of prognostic genes in LAC

COX regression method was performed to authenticate the influencing genes of OS, DSS and PFI in LAC patients, with *P* < 0.01 to define the prognostic genes of LAC. The intersection of the prognostic genes and SAAL1 co-expressed genes in LAC was obtained. The expression levels of the intersection genes CCNB1, SGO1, GTSE1, E2F7, MCM4, DLGAP5, CDCA2, CENPK, FAM111B, SPC24, DEPDC1B, CENPH, CDC25C, PARPBP, FANCI, SPC25, KIF18A, BUB1B, KIF20A, SGO2, and TTK in LAC tissues were obtained, and their relationship with living states in LAC patients was estimated using K-M survival and differential expression analysis.

### Immunohistochemical analyses

The tissues were gained from the Department of Pathology of our hospital to confirm the SAAL1 levels, which was estimated via the ethics committee of Wuhan Union Hospital. The tissues from patients were subjected to routine dewaxing and hydration, sodium citrate buffer antigen repair, blocking, incubation with anti-human SAAL1 antibody antibodies (1:200, Proteintech, China) and secondary antibodies, DAB color development, counterstaining, and sheet sealing. The relative expression levels of SAAL1 protein were calculated as described in a previous study [14].

### Construction of the cell model with down-regulation of SAAL1 expression

The A549 cells were cultivated in RPIM-1640 medium, and subcultured once every 2–3 days. A549 cells with good growth were placed in a 6-well plate, and sh-SAAL1 solution and pro-transfection reagent (sh-SAAL1 target sequence was 5'-CCACCUACUCUGCUGGAAATT-3', GeneChem, China) were added at 24 h to establish the LAC cell model with down-regulated SAAL1 expression. The expression levels of SAAL1 in the blank, control, and interference cell groups was identified via western blotting.

### EDU experiment

The EDU assay is an experimental technique for detecting apoptosis of cancer cells. After the establishment of the A549 cell model, the reagents were added according to the instructions of the EDU kit. Subsequently, DNA counterstaining, imaging and apoptosis analysis of A549 cells were performed [15]. The EDU experiment was repeated three times.

### The mechanisms of SAAL1 involvement in LAC progression

The data of LAC tissues obtained were classified into two groups via the SAAL1 median value. The effects of SAAL1 expression changes on gene sets were explored on the Gene set enrichment analysis (GSEA) software platform to determine the signaling mechanisms involved in LAC progression [16, 17]. NOM p-value was used as the significance standard. Based on the GSEA results, western blotting was used to determine the signaling pathways of SAAL1 involved in LAC progression.

### Western blotting

After the establishment of SAAL1 expression model cells, the A549 cells in the blank, control, and interference with SAAL1 expression groups were lysed by cell lysate, and BCA quantitative analysis was performed. Extracted proteins were resolved via the sodium dodecylsulfate-polyacrylamide gel electrophoresis and electro-transferred onto the polyvinylidene difluoride (PVDF) membrane. 1: 1000 SAAL1, cyclin D1, and Bcl-2 antibody (Proteintech, China) incubation was performed after the membranes of western blotting were cut. The membranes were washed, and secondary antibody incubation was completed. The membranes were washed again, and then the SAAL1, cyclin D1, and Bcl-2 protein levels in each group were calculated.

### The relationship between SAAL1 level and immune microenvironment

The Estimation of Stromal and Immune cells in Malignant Tumor tissues using Expression data (ESTIMATE) and single sample gene set enrichment analysis (ssGSEA) ways were performed to determine the immune scores and immune cells in LAC tissues. Moreover, correlation method was performed to identify the relation between the SAAL1 levels and the levels of immune scores, and immune cells.

### Statistical analysis

The Wilcoxon rank sum test and chi-square test were used to determine the SAAL1 levels in LAC tissues of TCGA database, and the t-test was used to determine the SAAL1 levels in clinical LAC tissues and cell models. ROC, COX and survival methods were carried out the diagnostic and prognostic values of SAAL1 expression. Correlation technique was carried out the relationship between SAAL1 levels and immune infiltration. *P* < 0.05 was known as the statistically significant.

## Results

### Significantly higher expression levels of SAAL1 were found in LAC

**Fig. 1:**
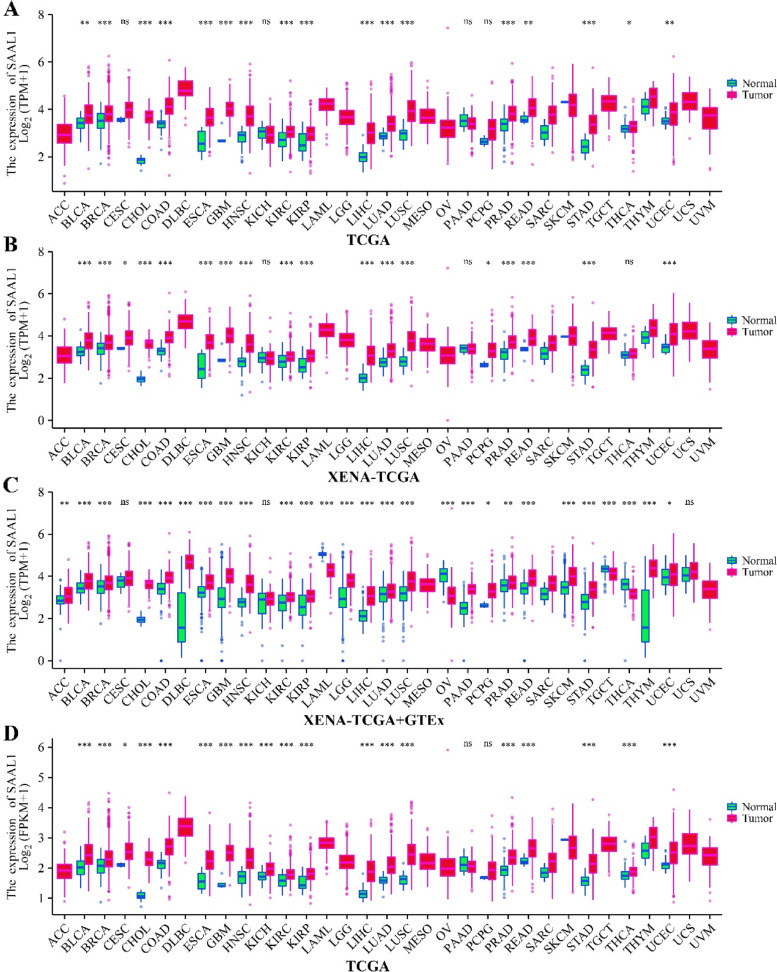
SAAL1 expression in unpaired pan-cancer tissues. **A** TCGA; **B** XENA-TCGA; **C** XENA-TCGA + GTEx; **D** TCGA. Note: TCGA, the cancer genome atlas; GTEx, Genotypic Tissue Expression

**Fig. 2:**
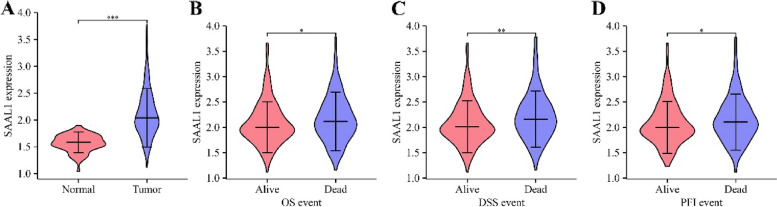
SAAL1 expression in LAC tissues. **A** Normal tissues vs LAC tissues; **B** OS; **C** DSS; **D** PFI. Note: OS, overall survival; PFI, progression-free interval; DSS, disease-specific interval; LAC, lung adenocarcinoma

SAAL1 was overexpressed in KIRP, HNSC, LUSC, BLCA, KICH, CESC, STAD, LIHC, COAD, LAC, KIRC, CHOL, PRAD, BRCA, READ, GBM, THCA, ESCA, UCEC, and others in the unpaired cancer patients (Fig. 1A-D), and in UCEC, BLCA, THCA, LIHC, PRAD, COAD, KICH, KIRC, HNSC, KIRP, CHOL, LUSC, ESCA, STAD, BRCA, LAC, and others in the paired cancer patients (Figure S1). SAAL1 was particularly overexpressed in LAC tissues (Fig. 2A).

### Overexpression of SAAL1 had something to do with diagnosis, T stage and dismal prognosis in LAC

**Fig. 3:**
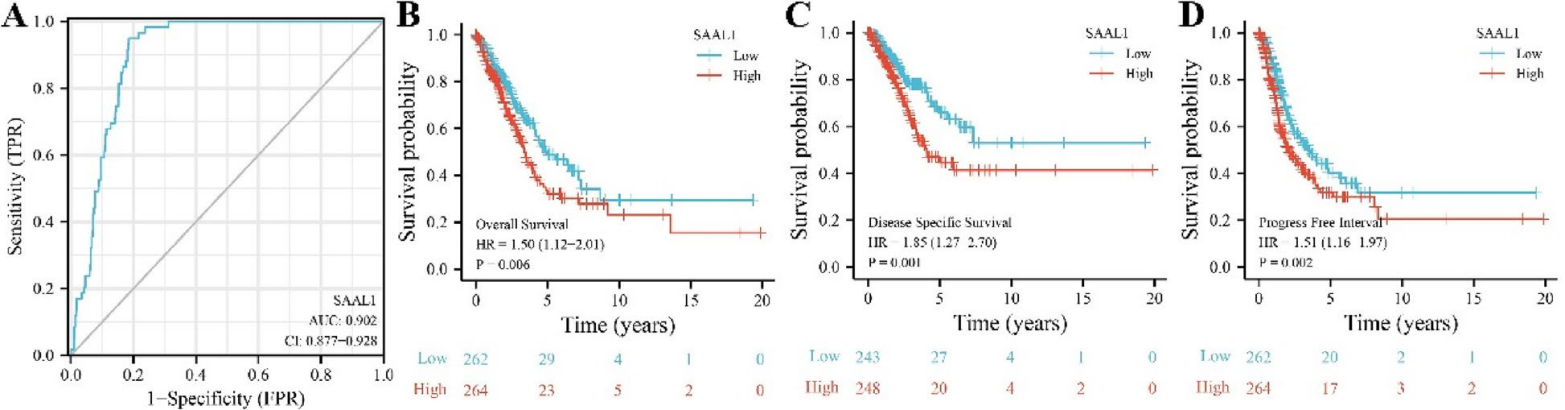
The diagnostic and prognostic values of SAAL1 expression in LAC. **A** ROC analysis; **B** OS; **C** DSS; **D** PFI. Note: OS, overall survival; PFI, progression-free interval; DSS, disease-specific survival; ROC, receiver operating characteristic; LAC, lung adenocarcinoma

Elevated SAAL1 expression levels were observed in tissues of deceased LAC patients (Fig. 2B-D). In addition, the high- and low- SAAL1 expression was related to the T stage and survival time of LAC patients, and has statistical significance (Table 1). Receiver operating characteristic (ROC) analysis showed that the area under the curve of SAAL1 in normal tissues and cancer tissues was 0.902 (Fig. 3A), indicating the potential diagnostic value of SAAL1 in LAC. K-M survival showed that SAAL1 overexpression had something to do with the short OS, DSS, and PFI in LAC patients (Fig. 3B-D). Univariate and multivariate COX regression analyses revealed that SAAL1 overexpression was an independent risk factor for OS, DSS and PFI in LAC patients (Tables 2, 3, 4).

**Table 1:** The relationship between high- and low- expression of SAAL1 and clinical characteristics of LAC patients

Characteristic	Low SAAL1 expression	High SAAL1 expression	*P*
N	267	268	
T stage			0.007
T1	101 (19%)	74 (13.9%)	
T2	124 (23.3%)	165 (31%)	
T3	29 (5.5%)	20 (3.8%)	
T4	11 (2.1%)	8 (1.5%)	
N stage			0.339
N0	178 (34.3%)	170 (32.8%)	
N1	49 (9.4%)	46 (8.9%)	
N2	32 (6.2%)	42 (8.1%)	
N3	0 (0%)	2 (0.4%)	
M stage			0.679
M0	179 (46.4%)	182 (47.2%)	
M1	14 (3.6%)	11 (2.8%)	
Pathologic stage			0.200
Stage I	147 (27.9%)	147 (27.9%)	
Stage II	67 (12.7%)	56 (10.6%)	
Stage III	34 (6.5%)	50 (9.5%)	
Stage IV	15 (2.8%)	11 (2.1%)	
Primary therapy outcome			0.212
PD	30 (6.7%)	41 (9.2%)	
SD	17 (3.8%)	20 (4.5%)	
PR	5 (1.1%)	1 (0.2%)	
CR	168 (37.7%)	164 (36.8%)	
Gender			0.631
Female	146 (27.3%)	140 (26.2%)	
Male	121 (22.6%)	128 (23.9%)	
Race			0.146
Asian	6 (1.3%)	1 (0.2%)	
Black or African American	25 (5.3%)	30 (6.4%)	
White	199 (42.5%)	207 (44.2%)	
Age			0.596
< = 65	123 (23.8%)	132 (25.6%)	
> 65	133 (25.8%)	128 (24.8%)	
Anatomic neoplasm subdivision			0.142
Left	110 (21.2%)	95 (18.3%)	
Right	147 (28.3%)	168 (32.3%)	
Smoker			0.630
No	40 (7.7%)	35 (6.7%)	
Yes	221 (42.4%)	225 (43.2%)	
OS event			0.003
Alive	188 (35.1%)	155 (29%)	
Dead	79 (14.8%)	113 (21.1%)	
DSS event			< 0.001
Alive	205 (41.1%)	174 (34.9%)	
Dead	43 (8.6%)	77 (15.4%)	
PFI event			0.007
Alive	170 (31.8%)	139 (26%)	
Dead	97 (18.1%)	129 (24.1%)	

Note: *LAC* Lung adenocarcinoma, *OS* Overall survival, *PFI* Progression-free interval, *DSS* Disease-specific survival

**Table 2:** Cox regression analysis revealed the risk factors for OS in LAC patients

Characteristics	*N*	Univariate analysis		Multivariate analysis	
		HR (95% CI)	*P*	HR (95% CI)	*P*
T stage	523				
T1	175	Reference			
T2	282	1.521 (1.068–2.166)	0.020	1.318 (0.830–2.092)	0.242
T3	47	2.937 (1.746–4.941)	< 0.001	3.078 (1.525–6.214)	0.002
T4	19	3.326 (1.751–6.316)	< 0.001	2.329 (1.040–5.219)	0.040
N stage	510				
N0	343	Reference			
N1	94	2.381 (1.695–3.346)	< 0.001	2.436 (1.275–4.655)	0.007
N2	71	3.108 (2.136–4.521)	< 0.001	2.270 (0.953–5.403)	0.064
N3	2	0.000 (0.000-Inf)	0.994	0.000 (0.000-Inf)	0.993
M stage	377				
M0	352	Reference			
M1	25	2.136 (1.248–3.653)	0.006	2.059 (0.944–4.492)	0.070
Pathologic stage	518				
Stage I	290	Reference			
Stage II	121	2.418 (1.691–3.457)	< 0.001	0.979 (0.487–1.969)	0.954
Stage III	81	3.544 (2.437–5.154)	< 0.001	1.342 (0.511–3.524)	0.550
Stage IV	26	3.790 (2.193–6.548)	< 0.001		
Gender	526				
Female	280	Reference			
Male	246	1.070 (0.803–1.426)	0.642		
Age	516				
< = 65	255	Reference			
> 65	261	1.223 (0.916–1.635)	0.172		
SAAL1	526				
Low	262	Reference			
High	264	1.500 (1.123–2.005)	0.006	1.890 (1.314–2.719)	< 0.001

Note: *LAC* Lung adenocarcinoma, *OS* Overall survival

**Table 3:** Cox regression analysis revealed the risk factors for DSS in LAC patients

Characteristics	*N*	Univariate analysis		Multivariate analysis	
		HR (95% CI)	*P*	HR (95% CI)	*P*
T stage	488				
T1	168	Reference			
T2	262	1.701 (1.085–2.668)	0.021	1.319 (0.738–2.359)	0.350
T3	43	2.846 (1.453–5.572)	0.002	2.771 (1.070–7.174)	0.036
T4	15	2.770 (1.061–7.230)	0.037	2.691 (0.822–8.811)	0.102
N stage	475				
N0	327	Reference			
N1	83	2.751 (1.808–4.185)	< 0.001	2.669 (1.151–6.188)	0.022
N2	63	2.762 (1.698–4.493)	< 0.001	2.605 (0.751–9.030)	0.131
N3	2	0.000 (0.000-Inf)	0.995	0.000 (0.000-Inf)	0.996
M stage	344				
M0	323	Reference			
M1	21	2.455 (1.269–4.749)	0.008	2.719 (1.127–6.555)	0.026
Pathologic stage	483				
Stage I	277	Reference			
Stage II	112	3.017 (1.931–4.715)	< 0.001	1.033 (0.417–2.558)	0.945
Stage III	72	3.326 (2.028–5.457)	< 0.001	0.988 (0.247–3.943)	0.986
Stage IV	22	4.632 (2.371–9.050)	< 0.001		
Gender	491				
Female	262	Reference			
Male	229	0.989 (0.687–1.424)	0.954		
Age	481				
< = 65	243	Reference			
> 65	238	1.013 (0.701–1.464)	0.944		
SAAL1	491				
Low	243	Reference			
High	248	1.850 (1.270–2.696)	0.001	2.520 (1.559–4.073)	< 0.001

Note: *LAC* Lung adenocarcinoma, *DSS* Disease-specific survival

**Table 4:** Cox regression analysis revealed the risk factors for PFI in LAC patients

Characteristics	*N*	Univariate analysis		Multivariate analysis	
		HR (95% CI)	*P*	HR (95% CI)	*P*
T stage	523				
T1	175	Reference			
T2	282	1.758 (1.276–2.422)	< 0.001	1.428 (1.025–1.991)	0.035
T3	47	3.495 (2.199–5.556)	< 0.001	2.593 (1.419–4.738)	0.002
T4	19	1.113 (0.444–2.791)	0.819	0.590 (0.190–1.834)	0.362
N stage	510				
N0	343	Reference			
N1	94	1.540 (1.118–2.122)	0.008	1.215 (0.692–2.135)	0.497
N2	71	1.498 (1.018–2.205)	0.040	0.560 (0.192–1.633)	0.288
N3	2	0.906 (0.127–6.485)	0.922	0.546 (0.062–4.845)	0.587
M stage	377				
M0	352	Reference			
M1	25	1.513 (0.855–2.676)	0.155		
Pathologic stage	518				
Stage I	290	Reference			
Stage II	121	2.013 (1.478–2.742)	< 0.001	1.404 (0.795–2.479)	0.242
Stage III	81	1.831 (1.257–2.669)	0.002	2.608 (0.841–8.085)	0.097
Stage IV	26	2.086 (1.189–3.657)	0.010	2.091 (1.064–4.110)	0.032
Gender	526				
Female	280	Reference			
Male	246	1.172 (0.901–1.526)	0.236		
Age	516				
< = 65	255	Reference			
> 65	261	1.023 (0.784–1.335)	0.867		
SAAL1	526				
Low	262	Reference			
High	264	1.511 (1.159–1.970)	0.002	1.581 (1.195–2.091)	0.001

Note: LAC, lung adenocarcinoma; PFI, progression-free interval

### Increased expression of SAAL1 had something to do with poor existence values in patient subgroups

**Fig. 4:**
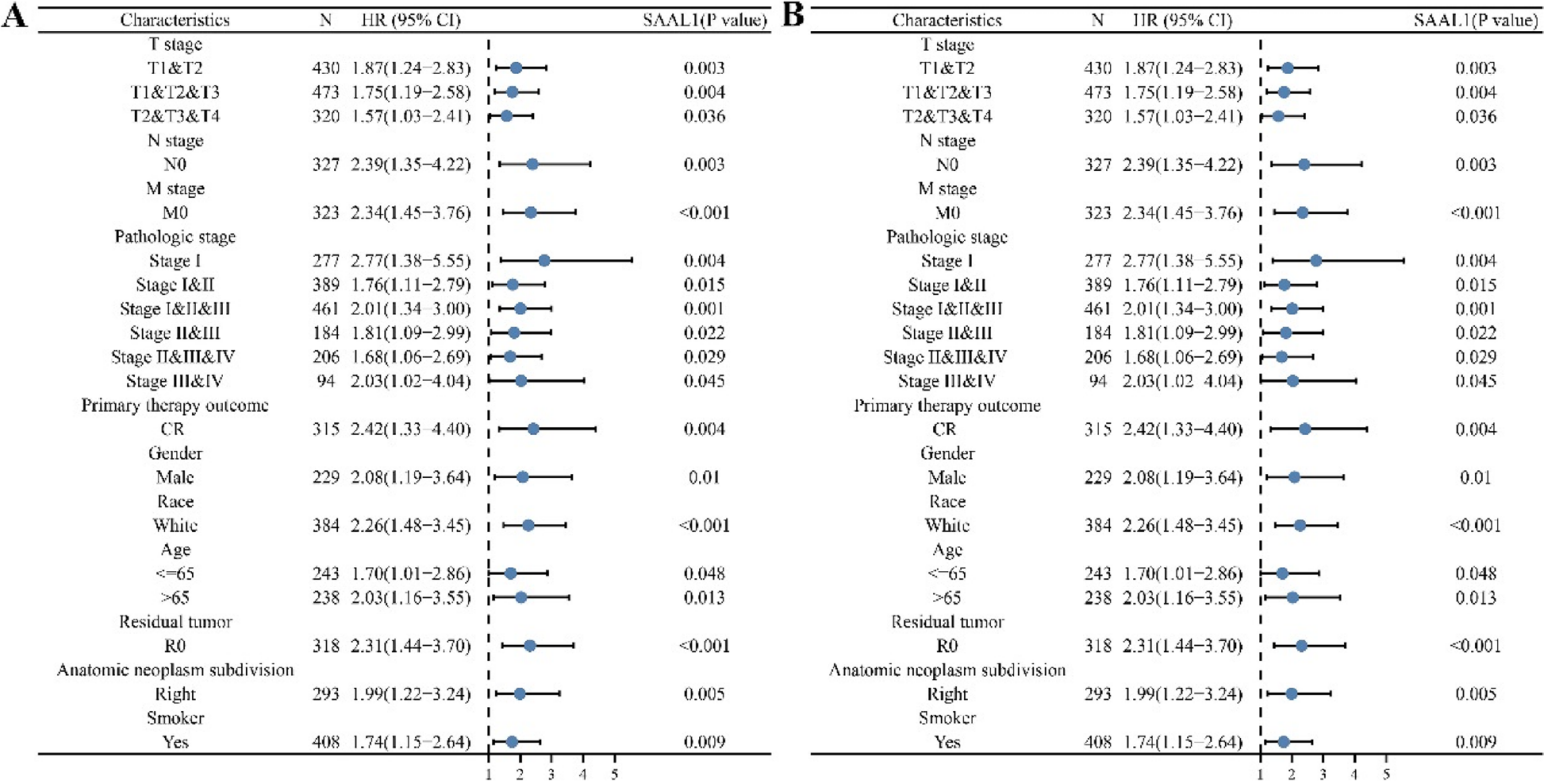
Increased SAAL1 expression was associated with poorer OS and DSS in LAC patient subgroups. **A** OS; **B** DSS. Note: OS, overall survival; LAC, lung adenocarcinoma; DSS, disease-specific survival

Increased SAAL1 expression was associated with shorter OS in LAC patients with demographic features stage T1-2, stage T1-3, N0, left lung, right lung, M0, pathological stage, CR, male, white, age (< = 65), RO, and smoking history (Fig. 4). In addition, increased SAAL1 level had something to do with shorter DSS in subgroups of LAC patients with stage T1-2, stage T1-3, white, stage T2-4, M0, pathological stage, N0, CR, male, right lung, age (> 65), RO, age (< = 65), and smoking history, as displayed in Fig. 4. Furthermore, higher SAAL1 level had something to do with shorter PFI in subgroups of LAC patients with demographic features stage T1, stage T1-2, stage T1-3, N0, stage T2, white, stage T2-4, left lung, right lung, M0, male, pathological stage, age (> 65), CR, female, RO, no smoking history, and smoking history (Figure S2).

### Functions and PPI network of SAAL1-associated genes

**Fig. 5:**
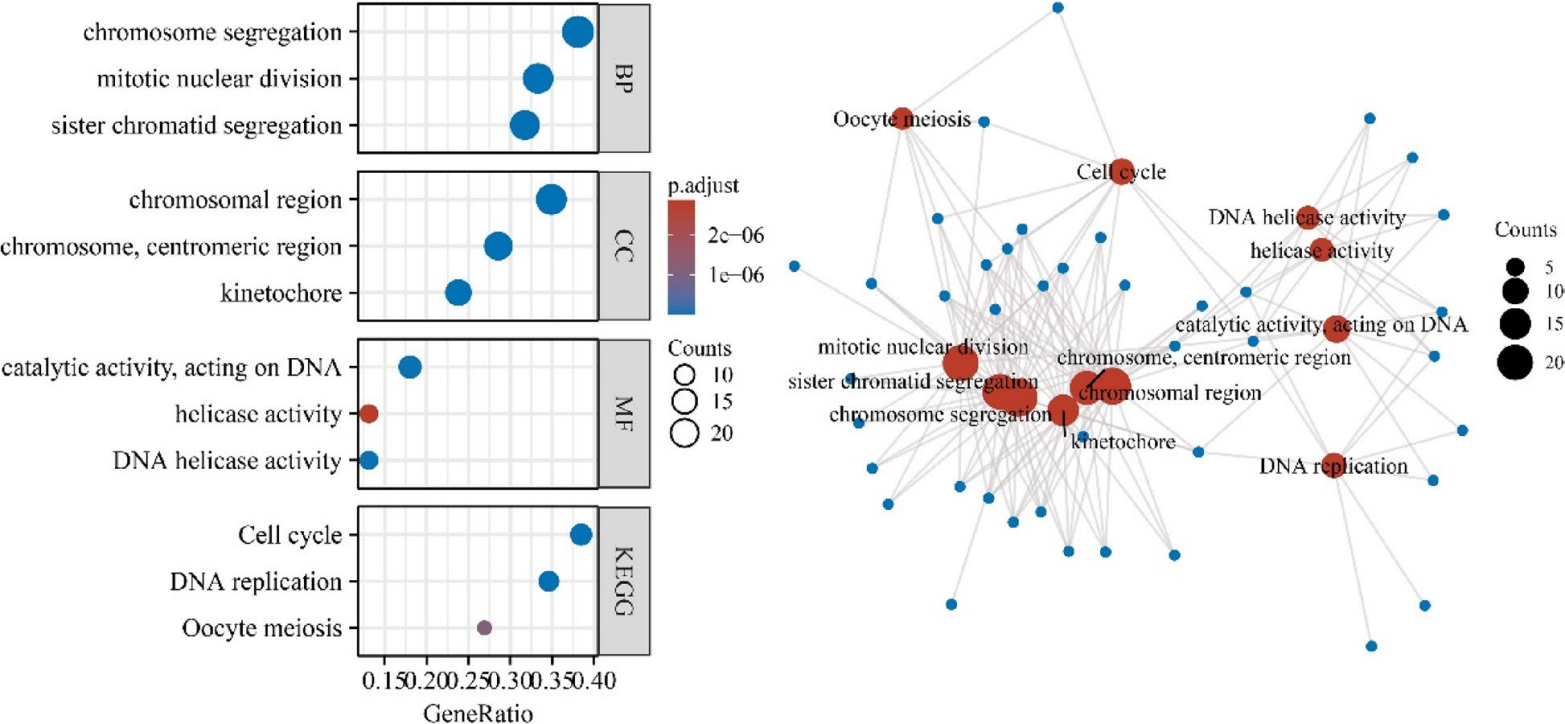
Functions and signaling pathways of SAAL1-associated genes

Based on the study criteria, 64 genes were identified as strongly related to SAAL1 (Table 5). SAAL1-associated genes were involved in segregation, mitotic nuclear division, nuclear division, DNA replication, cell cycle and meiotic cell cycle process, cell cycle G1/S phase transition, spindle checkpoint, positive regulation of cell cycle, DNA recombination, and others (Fig. 5 and Table S1). In terms of mechanisms, the SAAL1-related genes were enriched in base excision repair, DNA replication, cell cycle, mismatch repair, cellular senescence, and others (Fig. 5 and Table 6). Figure S3 illustrates the protein–protein interaction (PPI) network of the SAAL1-related genes.

**Table 5:** SAAL1-associated genes

Gene	r	*p*	Gene	r	*p*
NCAPG	0.552988531	3.54892E-44	DSCC1	0.532620346	1.56765E-40
DLGAP5	0.518746717	3.4648E-38	MELK	0.514004467	2.07283E-37
TTK	0.515605675	1.13662E-37	PSMA1	0.535849494	4.30361E-41
SKA1	0.541999372	3.52932E-42	RAD51AP1	0.531133944	2.82907E-40
CHEK1	0.529465685	5.46886E-40	VRK1	0.509401149	1.14576E-36
FANCG	0.523832118	4.93025E-39	E2F7	0.511543	5.18816E-37
RFC5	0.509099787	1.28029E-36	SPDL1	0.525544676	2.53781E-39
POLA2	0.501989002	1.70281E-35	PTTG1	0.50051243	2.8923E-35
TPX2	0.513378661	2.61923E-37	CCNB2	0.502227351	1.56287E-35
CDCA2	0.504811631	6.14062E-36	NCAPH	0.508551313	1.56653E-36
DDIAS	0.522759064	7.46012E-39	EIF3M	0.54806952	2.84063E-43
NDC80	0.528823382	7.04181E-40	GINS2	0.55147096	6.76676E-44
CENPH	0.563827448	3.20458E-46	CKAP2	0.517440556	5.68693E-38
CCDC34	0.519432655	2.66865E-38	LMNB1	0.547133619	4.20331E-43
FEN1	0.577025167	8.173E-49	KIFC1	0.500452946	2.95453E-35
SPC24	0.518715514	3.50615E-38	BUB1	0.524865117	3.30445E-39
GTSE1	0.506645554	3.14913E-36	MCM8	0.507542927	2.26796E-36
POLE2	0.514675755	1.61188E-37	SPC25	0.51846544	3.85567E-38
FAM111B	0.540135714	7.57186E-42	HASPIN	0.512203076	4.05959E-37
DEPDC1B	0.507983727	1.92955E-36	KIF20A	0.511583386	5.11096E-37
RNASEH2A	0.511086513	6.14541E-37	RRM1	0.623845835	4.86658E-59
CENPK	0.531492153	2.45455E-40	WDHD1	0.511076448	6.16838E-37
PRIM1	0.500704055	2.70052E-35	SGO2	0.507932879	1.96588E-36
MCM6	0.50150824	2.02396E-35	KIF18A	0.655641643	4.88088E-67
PRR11	0.500595374	2.80769E-35	FBXO5	0.528418122	8.25722E-40
CDC25C	0.521028815	1.45024E-38	MCM4	0.510832423	6.75204E-37
HJURP	0.505744469	4.37432E-36	KIF15	0.524586957	3.68087E-39
FANCI	0.529998723	4.43208E-40	SGO1	0.510301016	8.2192E-37
RFC4	0.519848327	2.27747E-38	GINS1	0.506599711	3.2023E-36
E2F8	0.522560379	8.05343E-39	PARPBP	0.503867524	8.64632E-36
PSMC3IP	0.512398745	3.77449E-37	CCNB1	0.514153033	1.96069E-37
CDCA5	0.533312577	1.18962E-40	BUB1B	0.507401847	2.38823E-36

**Table 6:** The mechanisms regulated by SAAL1-related genes

ID	Description	*P*	Count
hsa03030	DNA replication	6.97E-16	9
hsa04110	Cell cycle	2.18E-12	10
hsa04114	Oocyte meiosis	1.15E-07	7
hsa04115	p53 signaling pathway	7.90E-05	4
hsa04914	Progesterone-mediated oocyte maturation	0.000268389	4
hsa03420	Nucleotide excision repair	0.0004373	3
hsa03430	Mismatch repair	0.00241898	2
hsa05170	Human immunodeficiency virus 1 infection	0.004383701	4
hsa05166	Human T-cell leukemia virus 1 infection	0.004920727	4
hsa03410	Base excision repair	0.004949548	2
hsa03460	Fanconi anemia pathway	0.012870589	2
hsa04218	Cellular senescence	0.013271769	3
hsa03450	Non-homologous end-joining	0.041083372	1

### The prognostic roles of SAAL1-related genes in LAC

**Fig. 6:**
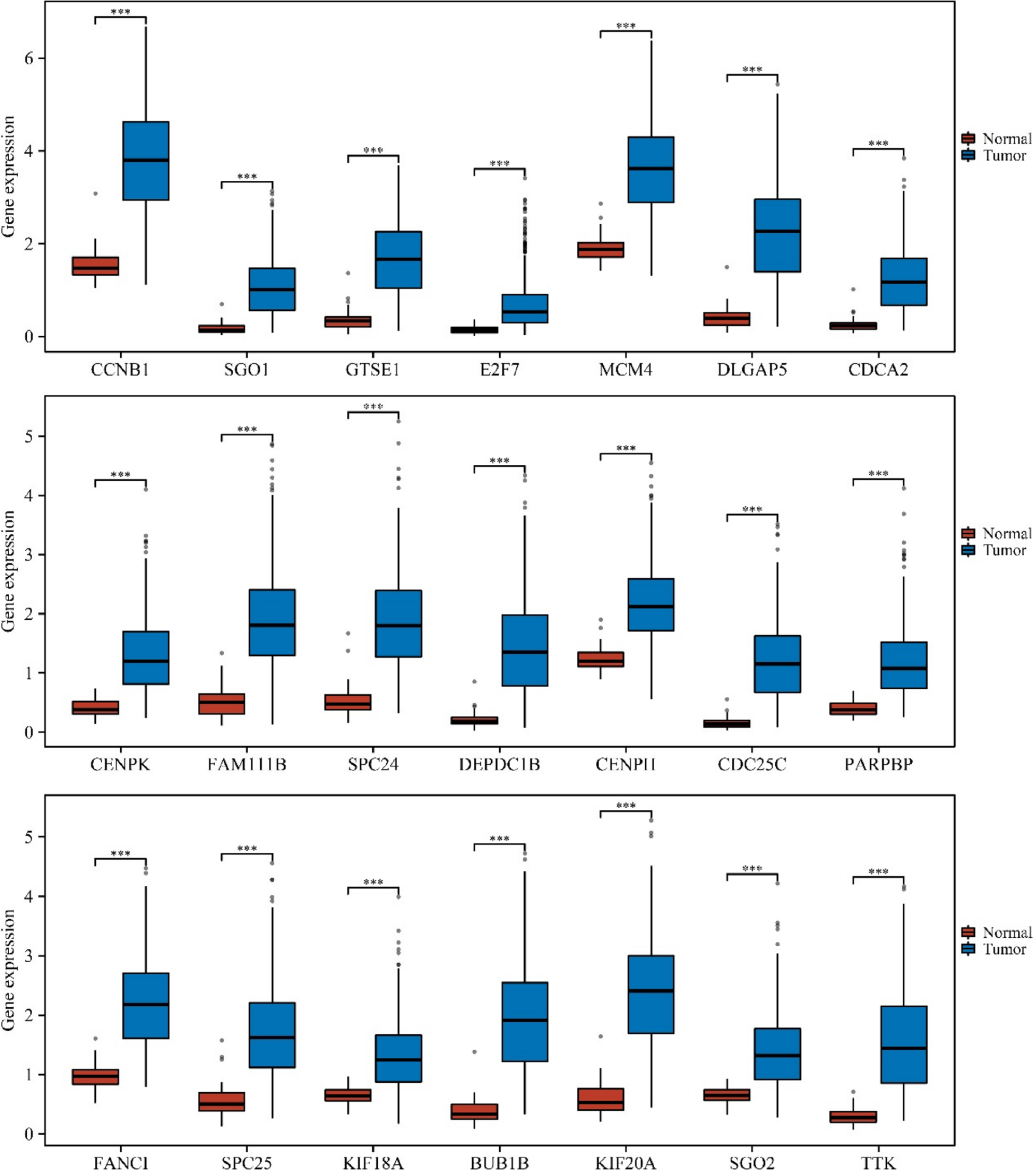
The expression levels of 21 SAAL1-related genes in LAC tissues. Note: LAC, lung adenocarcinoma

**Fig. 7:**
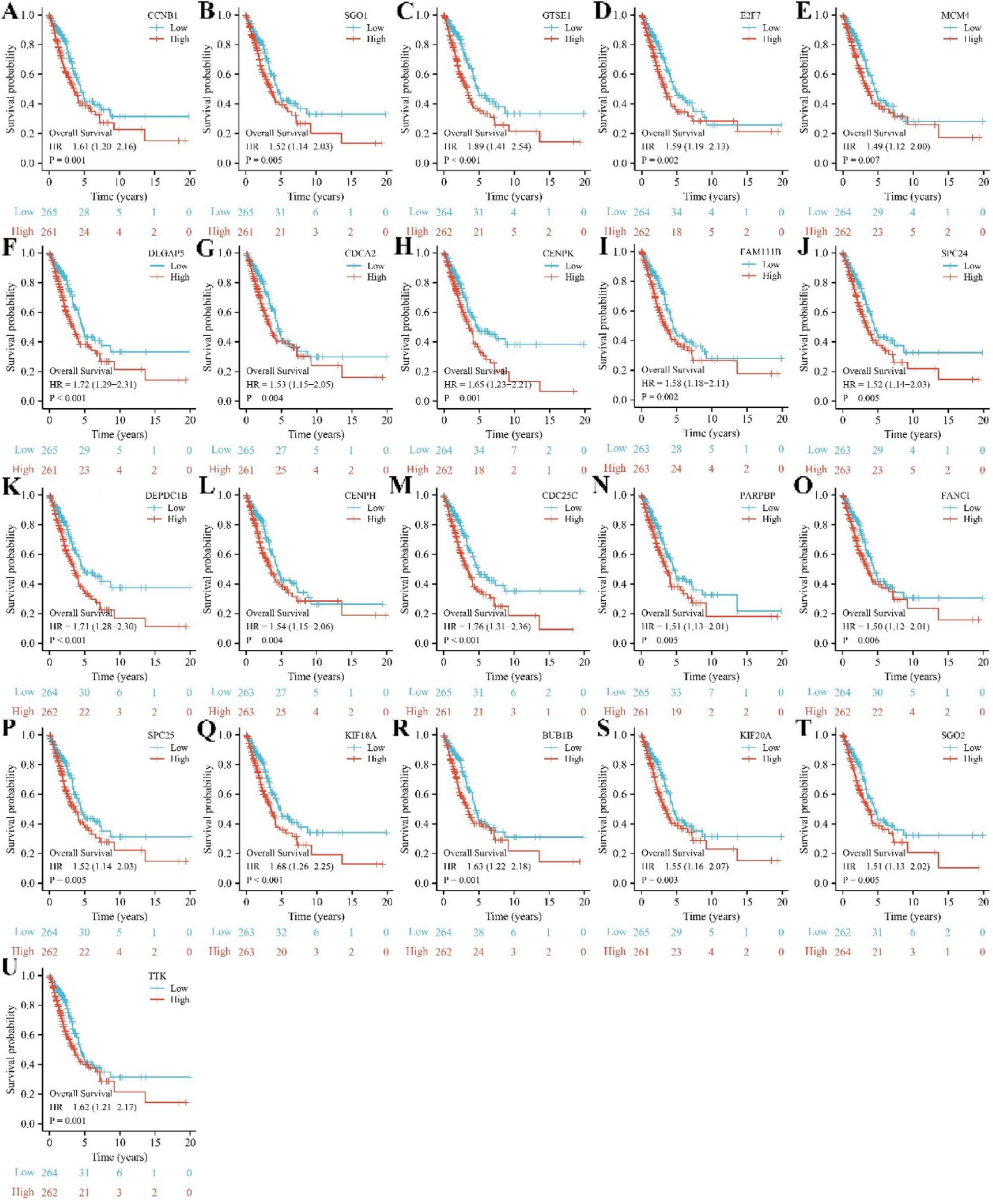
Increased SAAL1-related gene expression levels were associated with poorer OS in LAC patients. **A** CCNB1; **B** SGO1; **C** GTSE1; **D** E2F7; **E** MCM4; **F** DLGAP5; **G** CDCA2; **H** CENPK; **I** FAM111B; **J** SPC24; **K** DEPDC1B; **L** CENPH; **M** CDC25C; **N** PARPBP; **O** FANCI; **P** SPC25; **Q** KIF18A; **R** BUB1B; **S** KIF20A; **T** SGO2; **U **TTK. Note: OS, overall survival; LAC, lung adenocarcinoma

**Fig. 8:**
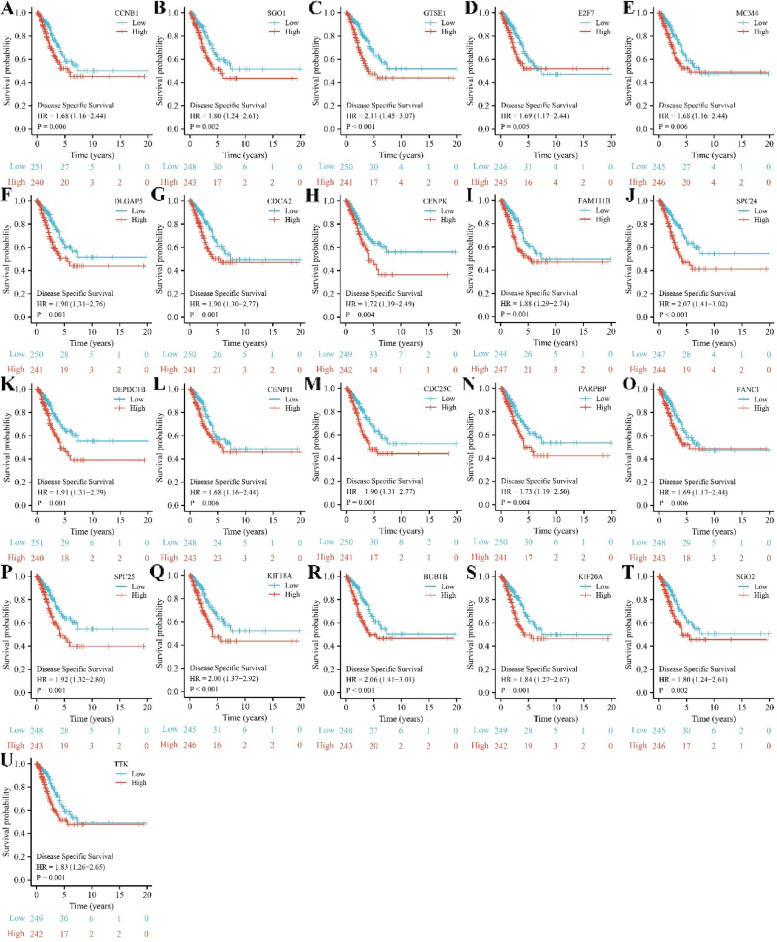
Increased SAAL1-related gene expression levels were associated with poorer DSS in LAC patients. **A** CCNB1; **B** SGO1; **C** GTSE1; **D** E2F7; **E** MCM4; **F** DLGAP5; **G** CDCA2; **H** CENPK; **I** FAM111B; **J** SPC24; **K** DEPDC1B; **L** CENPH; **M** CDC25C; **N** PARPBP; **O** FANCI; **P** SPC25; **Q** KIF18A; **R** BUB1B; **S** KIF20A; **T** SGO2; **U **TTK. Note: DSS, disease-specific survival; LAC, lung adenocarcinoma

**Fig. 9:**
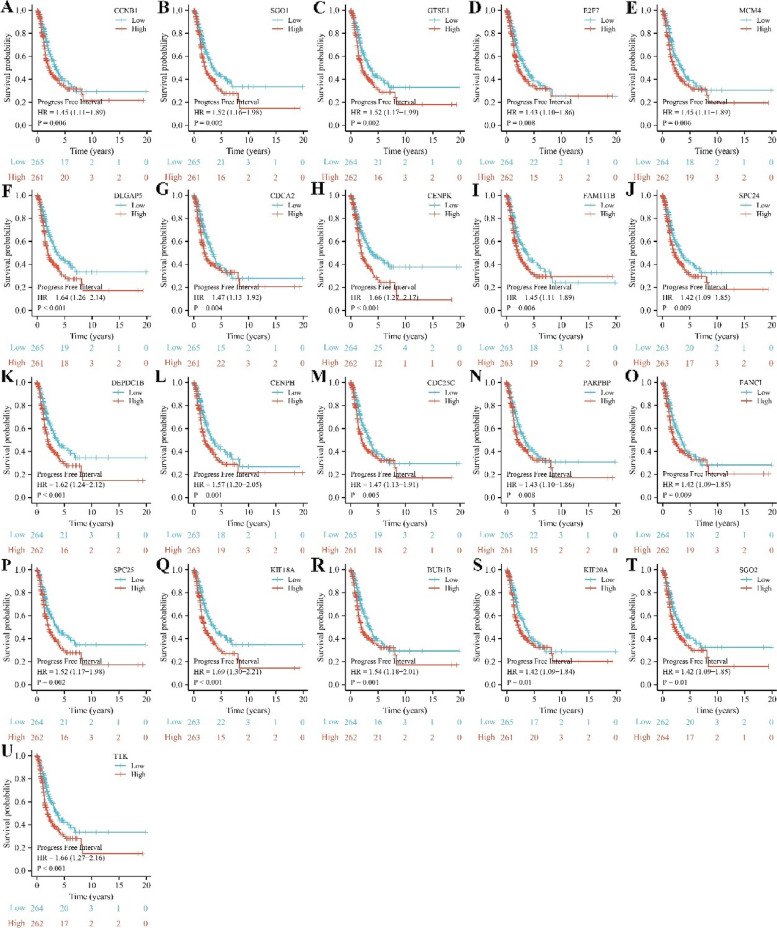
Increased SAAL1-related gene expression levels were associated with poorer PFI in LAC patients. **A** CCNB1; **B** SGO1; **C** GTSE1; **D** E2F7; **E** MCM4; **F** DLGAP5; **G** CDCA2; **H** CENPK; **I** FAM111B; **J** SPC24; **K** DEPDC1B; **L** CENPH; **M** CDC25C; **N** PARPBP; **O** FANCI; **P** SPC25; **Q** KIF18A; **R** BUB1B; **S**KIF20A; **T** SGO2; **U **TTK. Note: PFI, progression-free interval; LAC, lung adenocarcinoma

The intersection between prognostic factors of LAC patients and SAAL1-related genes included 21 genes. The expression levels of CCNB1, SGO1, GTSE1, E2F7, MCM4, DLGAP5, CDCA2, CENPK, FAM111B, SPC24, DEPDC1B, CENPH, CDC25C, PARPBP, FANCI, SPC25, KIF18A, BUB1B, KIF20A, SGO2, and TTK were significantly increased in unpaired and paired LAC tissues (Fig. 6 and S4). In addition, elevated CCNB1, SGO1, GTSE1, E2F7, MCM4, DLGAP5, CDCA2, CENPK, FAM111B, SPC24, DEPDC1B, CENPH, CDC25C, PARPBP, FANCI, SPC25, KIF18A, BUB1B, KIF20A, SGO2, and TTK expression levels had something to do with short OS, DSS, and PFI in LAC patients (Figs 7, 8, 9).

### Inhibition of SAAL1 expression could inhibit LAC growth by regulating the cell cycle

**Fig. 10:**
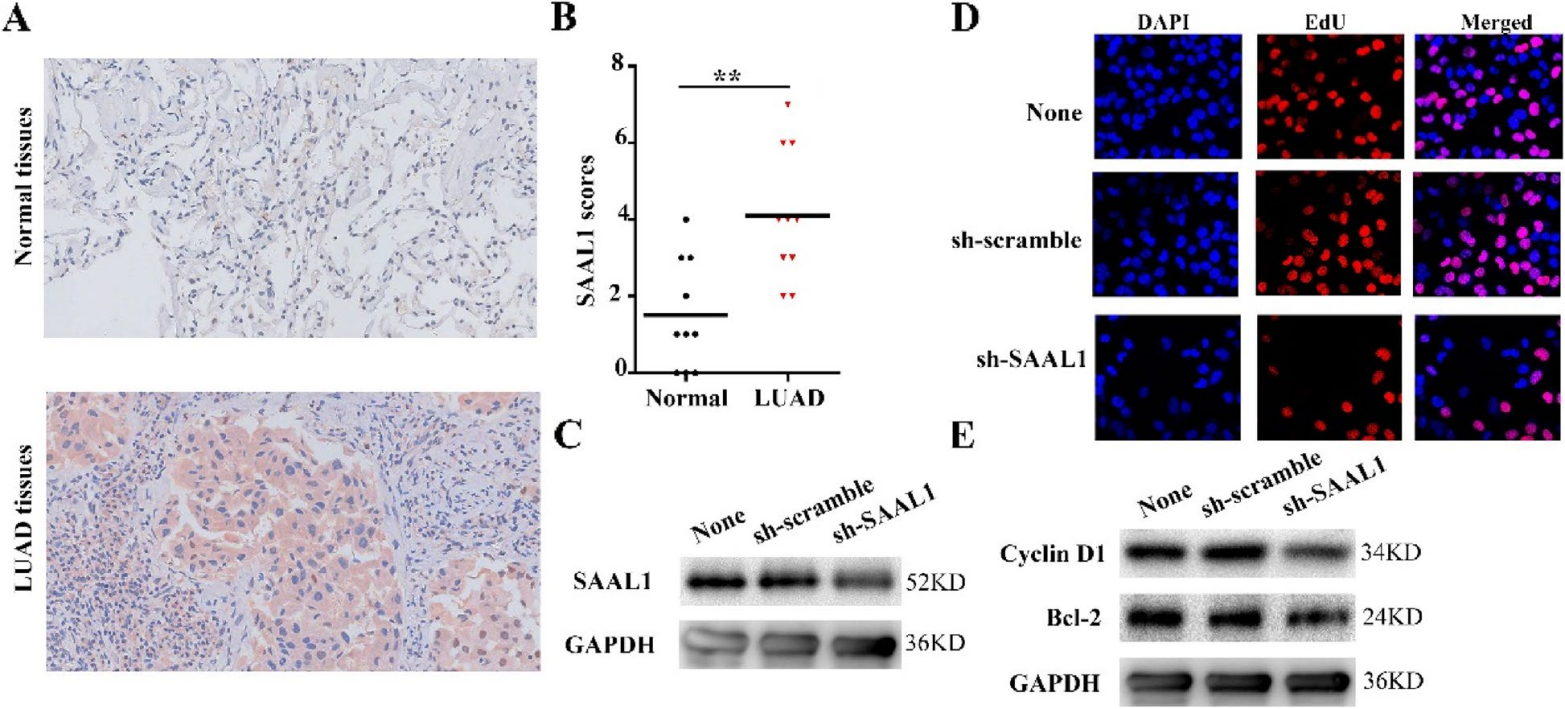
Inhibition of SAAL1 expression could inhibit LAC growth by regulating the cell cycle. (**A**-**B**) The expression levels in clinical tissues via IHC at 20 × magnification; **C** Identification of the A549 cell model with SAAL1 down-expression; **D** Apoptosis via EDU at 20 × magnification; **E** Cyclin D1 and Bcl-2 expression in the A549 cell model. Note: LAC, lung adenocarcinoma; **, *P* < 0.01

The IHC results revealed SAAL1 overexpression in LAC tissues (Fig. 10A-B). The GO annotation results demonstrated that the SAAL1-related genes were related to cell growth. Subsequently, a cell model with down-regulated SAAL1 expression was established by using shRNA interference technology. Inhibiting SAAL1 expression in LAC cells promoted cancer cell apoptosis (Fig. 10C-D). Moreover, mechanism analysis showed that SAAL1 was enriched in the cell cycle, DNA replication, and the p53 signaling (Table 7). Cyclin D1 and Bcl-2 genes were hub members of the cell cycle. We found that inhibition of SAAL1 expression resulted in significant down-regulation of cyclin D1 and Bcl-2 protein levels in A549 cells (Fig. 10E).

**Table 7:** The mechanisms impacted by SAAL1 over-expression

Name	Size	NES	NOM P
Cell cycle	124	2.3955967	0
Spliceosome	126	2.306933	0
Homologous recombination	28	2.2579896	0
RNA degradation	57	2.239501	0
Pyrimidine metabolism	97	2.1597848	0
Purine metabolism	156	2.1199543	0
Nucleotide excision repair	44	2.1095386	0.001976285
Oocyte meiosis	112	2.0554802	0
DNA replication	36	2.0520623	0
Mismatch repair	23	2.0502622	0.00203666
Basal transcription factors	35	2.0111003	0
Base excision repair	33	1.9896193	0.004048583
Progesterone mediated oocyte maturation	85	1.9445288	0
P53 signaling pathway	68	1.9198885	0.002066116
One carbon pool by folate	17	1.9191456	0.002040816
Ubiquitin mediated proteolysis	133	1.8948809	0
Proteasome	44	1.7589222	0.018480493
Aminoacyl tRNA biosynthesis	22	1.6986598	0.019646365
RNA polymerase	28	1.5847529	0.042596348
Cysteine and methionine metabolism	34	1.5472888	0.042424243

### SAAL1 expression had something to do with LAC immune microenvironment

**Fig. 11:**
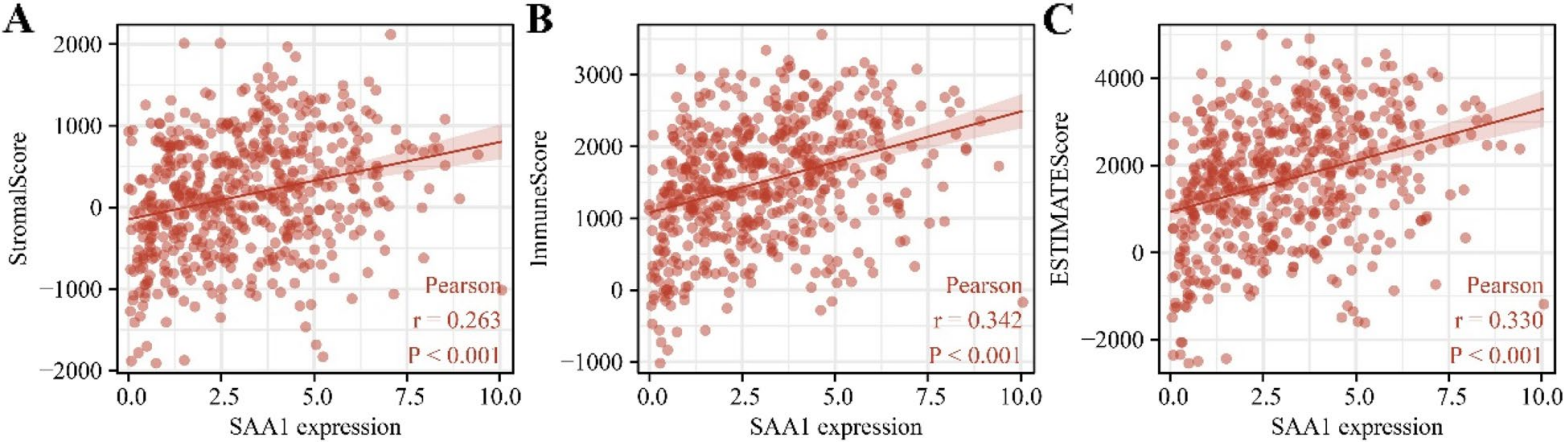
SAAL1 expression levels were significantly correlated with the stromal, estimate, and immune scores. **A** Stromal score; **B** Immune score; **C** Estimate score

Correlation method showed that the SAAL1 levels in LAC had something to do with the stromal score (0.263), estimate score (0.33), and immune score (0.342), and were related to the neutrophils (0.469), Th1 cells (0.334), pDC (0.304), cytotoxic cells (0.293), aDC (0.284), TReg (0.279), macrophages (0.227), T cells (0.224), B cells (0.222), NK CD56dim cells (0.31), Th2 cells (0.213), NK Cells (0.192), iDC (0.182), DC (0.173), NK CD56bright cells (0.171), Tgd (0.153), CD8 T Cells (0.135), Tcm (-0.126), eosinophils (0.12), and TFH (0.102) levels (Fig. 11, Figure S5 and Table 8).

**Table 8:** SAAL1 expression was correlated with LAC immune cells

Immune cells	r	*P*
aDC	0.284	< 0.001
B cells	0.222	< 0.001
CD8 T cells	0.135	0.002
Cytotoxic cells	0.293	< 0.001
DC	0.173	< 0.001
Eosinophils	0.120	0.006
iDC	0.182	< 0.001
Macrophages	0.227	< 0.001
Mast cells	0.035	0.420
Neutrophils	0.469	< 0.001
NK CD56bright cells	0.171	< 0.001
NK CD56dim cells	0.310	< 0.001
NK cells	0.192	< 0.001
pDC	0.304	< 0.001
T cells	0.224	< 0.001
T helper cells	0.023	0.601
Tcm	-0.126	0.004
Tem	0.066	0.125
TFH	0.102	0.018
Tgd	0.153	< 0.001
Th1 cells	0.334	< 0.001
Th17 cells	0.075	0.085
Th2 cells	0.213	< 0.001
TReg	0.279	< 0.001

Note: *LAC* Lung adenocarcinoma

## Discussion

Several research reports that inhibition of oncogenes or promotion of tumor suppressor gene expression could delay cancer progression and may improve the survival times of cancer patients [18–21]. Recent studies have discovered the overexpression of the inflammatory gene SAAL1 in HCC. Furthermore, elevated SAAL1 expression was had something to do with shorter OS in patients with HCC [8, 22]. Therefore, inhibition of SAAL1 expression could inhibit the growth and migration ability of HCC cells, impair the AKT/mTOR phosphorylation cascade driven by HGF/MET, and increase the sensitivity of HCC cells to sorafenib and foretinib [8]. This study demonstrated similar findings in LAC. SAAL1 was overexpressed in LAC tissues, with an area under the curve of 0.902, indicating the diagnostic potential of SAAL1 in LAC. SAAL1 overexpression had something to do with shorter OS, DSS, and PFI in LAC patients. COX regression analyses revealed that SAAL1 overexpression was an independent risk factor for OS, DSS and PFI in LAC patients. The results suggest that SAAL1 is a potential prognostic and diagnostic marker for LAC patients, which could be used as a target for LAC treatment.

The cell cycle was one of the key mechanisms of cancer cell growth [23–25]. For example, CCNB1 is an essential mitosis initiator and controller. Significant CCNB1 overexpression was observed in LAC patients and was correlated with tumor stage and shorter OS. Furthermore, CCNB1 overexpression may promote LAC cell proliferation, migration, invasion and cell cycle. In contrast, a negative correlation was found between miR-139-5p and CCNB1. miR-139-5p could reduce the expression level of CCNB1, thereby inhibiting cell growth and migration [23]. This study found that SAAL1 overexpression was associated with cell cycle, oocyte meiosis, DNA replication, and mismatch repair. Down-regulated expression of SAAL1 induced A549 cell apoptosis and inhibited the expression of cell cycle-related proteins cyclin D1 and Bcl-2, suggesting that SAAL1 could play an oncogene role in LAC progression, and promote LAC growth through cell cycle regulation.

In recent years, much research has focused on immunotherapy as a treatment option for LAC patients, which is expected to lengthen survival times [26–33]. For example, Huang et al. investigated 25 LAC patients treated with anti-PD-1/PD-L1 immunotherapy combined with anti-angiogenic agents and 49 LAC patients treated with anti-PD-1/PD-L1 monotherapy. The disease control rate, median progression-free survival (PFS), and median OS were significantly higher in the anti-PD-1/PD-L1 immunotherapy group compared to the anti-PD-1/PD-L1 monotherapy group. Moreover, the risk model revealed that anti-PD-1/PD-L1 immunotherapy combined with anti-angiogenesis prolonged the PFS in LAC patients [26]. Alausa et al. reported that G protein subunit gamma 12 (GNG12) silencing could activate the NF-kB signaling, inhibit the PD-L1 gene transcription, promote cancer immune escape, and then activate cancer proliferation, angiogenesis and immunotherapy resistance [30]. In this study, we found that the SAAL1 expression levels were correlated with stromal, immune, and estimate scores, and were correlated with immune cells, such as macrophages, T cells, B cells, Th2 cells, cytotoxic cells, and CD8 T cells. This indicated a significant correlation between SAAL1 expression and the LAC immune microenvironment.

In this research, we confirmed the role of SAAL1 in LAC progression by utilizing bioinformatics analysis and basic research. The findings of the present study reflect the results of Chu et al. in HCC [8, 22], indicating that SAAL1 could play an oncogenic role in LAC progression. However, this study has the following deficiencies: 1) we should further collect the tissue samples from LAC patients to detect SAAL1 expression levels, and explore the relationship between SAAL1 expression and clinical data of LAC patients to evaluate the clinical values of SAAL1; 2) we will add more LAC cell lines to verify the roles and mechanisms of SALL1 in vivo and in vitro. Overall, SAAL1 was overexpressed in LAC tissues, which was significantly associated with shorter OS, DSS, and PFI, and demonstrated diagnostic value for LAC patients. Inhibition of SAAL1 expression could inhibit the growth of LAC cells by regulating the cell cycle. SAAL1 overexpression was significantly correlated with the stromal score, immune score, estimate score, and immune infiltrating cell levels in LAC, which could be a new candidate target molecule for the treatment of LAC patients.

## Conclusions

SAAL1 is overexpressed in LUAD and is associated with poor survival of cancer patients. Inhibition of SAAL1 expression could regulate cancer growth via the cell cycle. This shows that SAAL1 is a promising prognostic biomarker in LAC patients.

## Supplementary Information

**Additional file 1.**

**Additional file 2 Figure S1.** SAAL1 expression in paired pan-cancer tissues. (A) TCGA; (B) XENA-TCGA; (C) TCGA. Note: TCGA, the cancer genome atlas. **Figure S2.** Increased SAAL1 expression was associated with poorer PFI in LAC patient subgroups. Note: PFI, progression-free interval; LAC, lung adenocarcinoma. **Figure S3.** PPI network of SAAL1-associated genes. Note: PPI, protein-protein interaction. **Figure S4.** The expression levels of 21 SAAL1-related genes in paired LAC tissues. Note: LAC, lung adenocarcinoma. **Figure S5.** SAAL1 expression levels were significantly correlated with the immune cells. (A) Neutrophils; (B) Th1 cells; (C) NK CD56dim cells; (D) pDC; (E) Cytotoxic cells; (F) aDC; (G) TReg; (H) Macrophages; (I) T cells. Table S1. Functions of SAAL1-associated genes.

## Data Availability

The data could be obtained from the corresponding author upon reasonable request.

## Abbreviations

SAAL1: Serum Amyloid A-like 1
LAC: Lung adenocarcinoma
OS: Overall survival
EMT: Epithelial-mesenchymal transition
CT: Chest computed tomography
PFI: Progression-free interval
GTEx: Genotypic Tissue Expression
HCC: Hepatocellular carcinoma
DSS: Disease-specific survival
GGN: Ground-glass nodules
TCGA: The Cancer Genome Atlas
