# Supplementary figures and images for: The effects of the prognostic biomarker SAAL1 on cancer growth and its association with the immune microenvironment in lung adenocarcinoma
Source: BMC Cancer. 2023 Mar 27;23:275. doi: 10.1186/s12885-023-10741-5 (PMC10041717; doi:10.1186/s12885-023-10741-5)

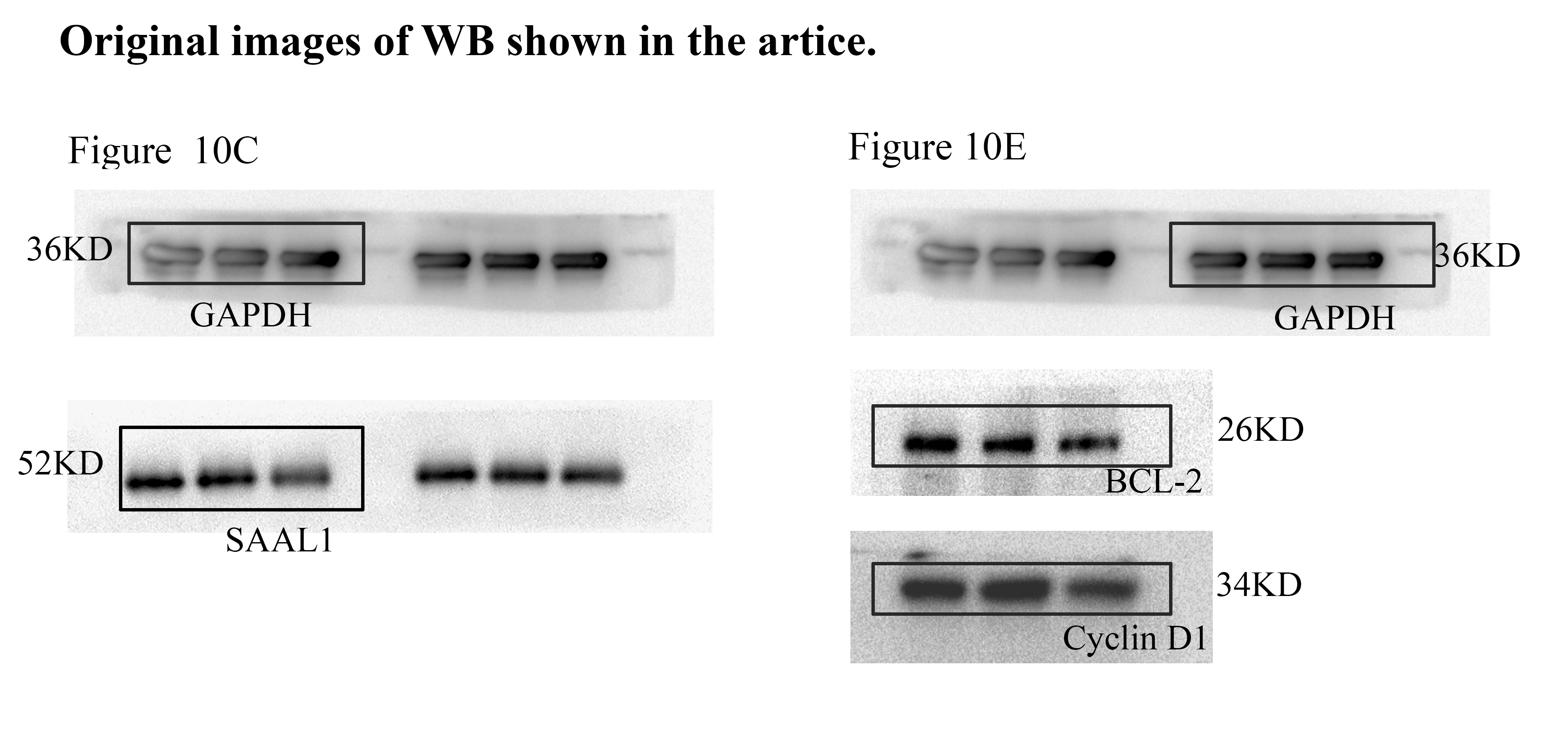

Supplement: Supplementary file 1 — Additional file 1. [file 12885_2023_10741_MOESM1_ESM.tif]
