# Supplementary material for: The effects of the prognostic biomarker SAAL1 on cancer growth and its association with the immune microenvironment in lung adenocarcinoma
Source: BMC Cancer. 2023 Mar 27;23:275. doi: 10.1186/s12885-023-10741-5 (PMC10041717; doi:10.1186/s12885-023-10741-5)
Supplement: Supplementary file 2 — Additional file 2 Figure S1. SAAL1 expression in paired pan-cancer tissues. (A) TCGA; (B) XENA-TCGA; (C) TCGA. Note: TCGA, the cancer genome atlas. Figure S2. Increased SAAL1 expression was associated with poorer PFI in LAC patient subgroups. Note: PFI, progression-free interval; LAC, lung adenocarcinoma. Figure S3. PPI network of SAAL1-associated genes. Note: PPI, protein-protein interaction. Figure S4. The expression levels of 21 SAAL1-related genes in paired LAC tissues. Note: LAC, lung adenocarcinoma. Figure S5. SAAL1 expression levels were significantly correlated with the immune cells. (A) Neutrophils; (B) Th1 cells; (C) NK CD56dim cells; (D) pDC; (E) Cytotoxic cells; (F) aDC; (G) TReg; (H) Macrophages; (I) T cells. Table S1. Functions of SAAL1-associated genes. [file 12885_2023_10741_MOESM2_ESM.docx]

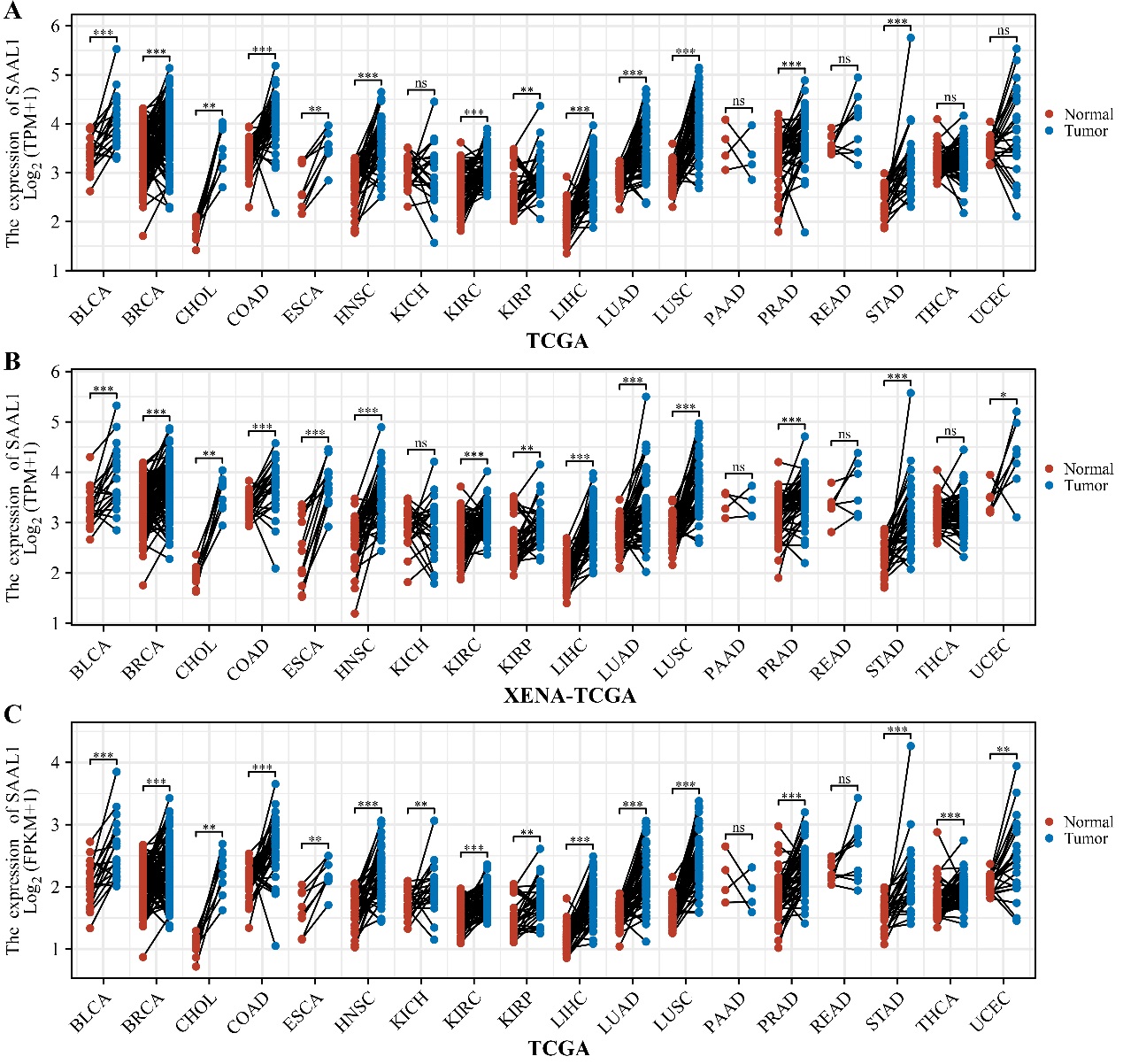


Figure S1. SAAL1 expression in paired pan-cancer tissues. (A) TCGA; (B) XENA-TCGA; (C) TCGA.

Note: TCGA, the cancer genome atlas.


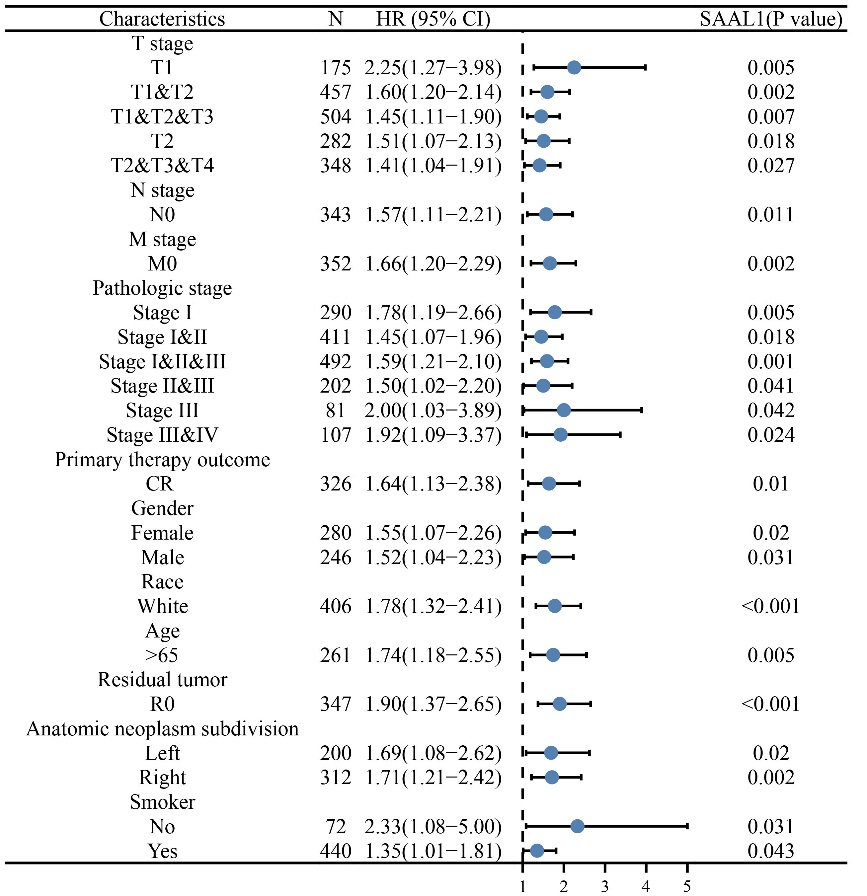


Figure S2. Increased SAAL1 expression was associated with poorer PFI in LAC patient subgroups.

Note: PFI, progression-free interval; LAC, lung adenocarcinoma.


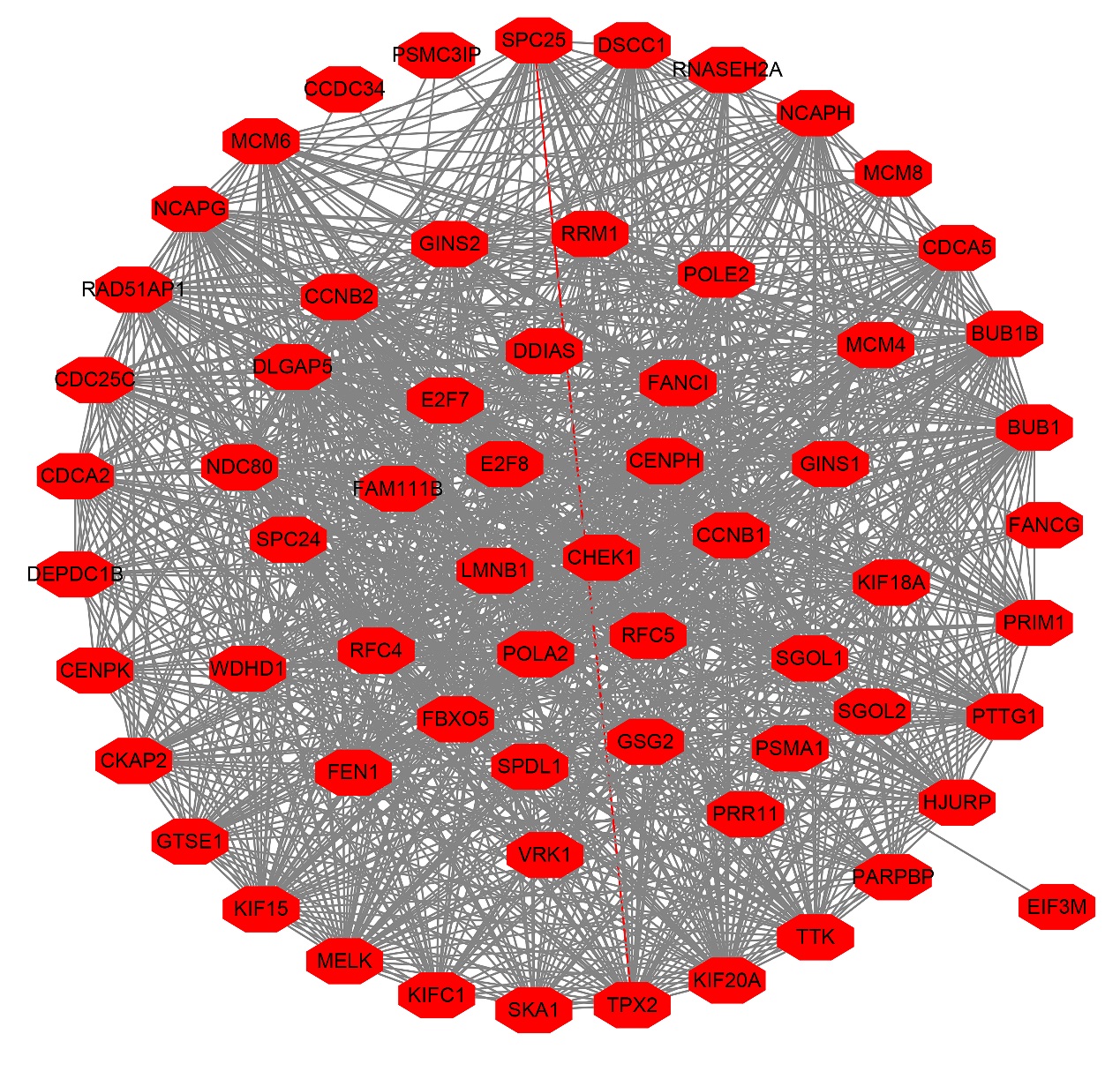


Figure S3. PPI network of SAAL1-associated genes.

Note: PPI, protein-protein interaction.


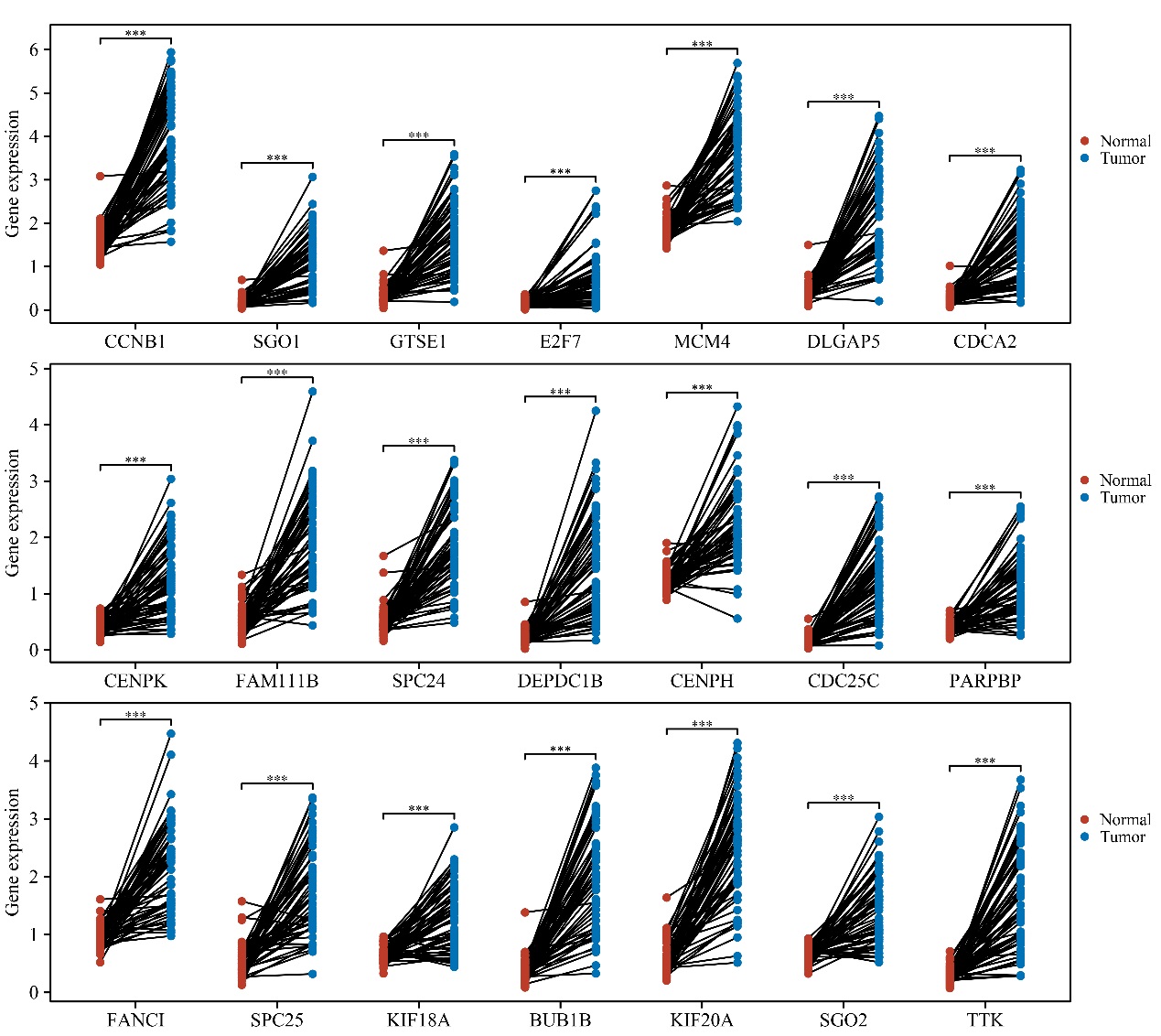


Figure S4. The expression levels of 21 SAAL1-related genes in paired LAC tissues.

Note: LAC, lung adenocarcinoma.


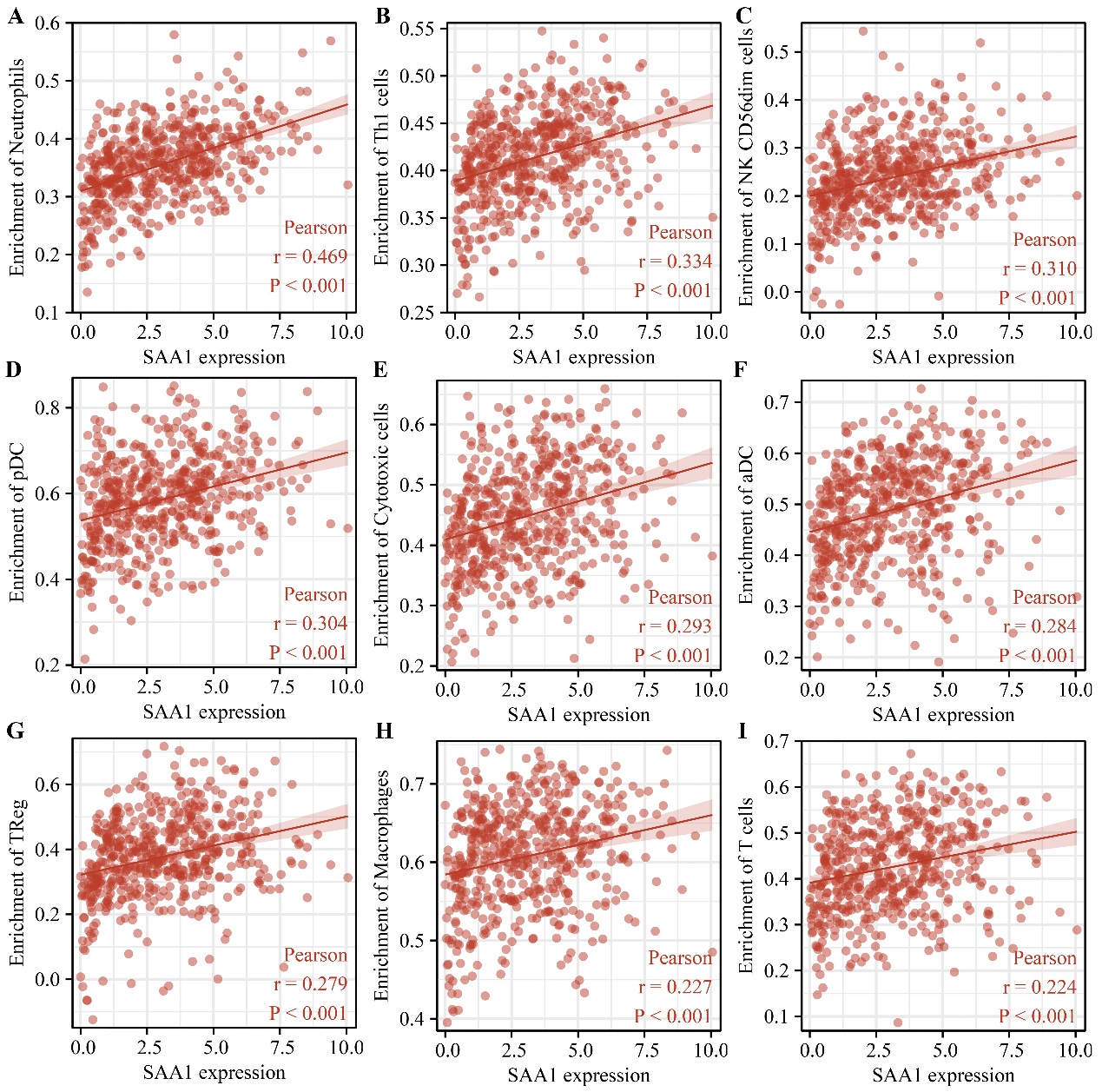


Figure S5. SAAL1 expression levels were significantly correlated with the immune cells. (A) Neutrophils; (B) Th1 cells; (C) NK CD56dim cells; (D) pDC; (E) Cytotoxic cells; (F) aDC; (G) TReg; (H) Macrophages; (I) T cells.

**Original images of western blotting**


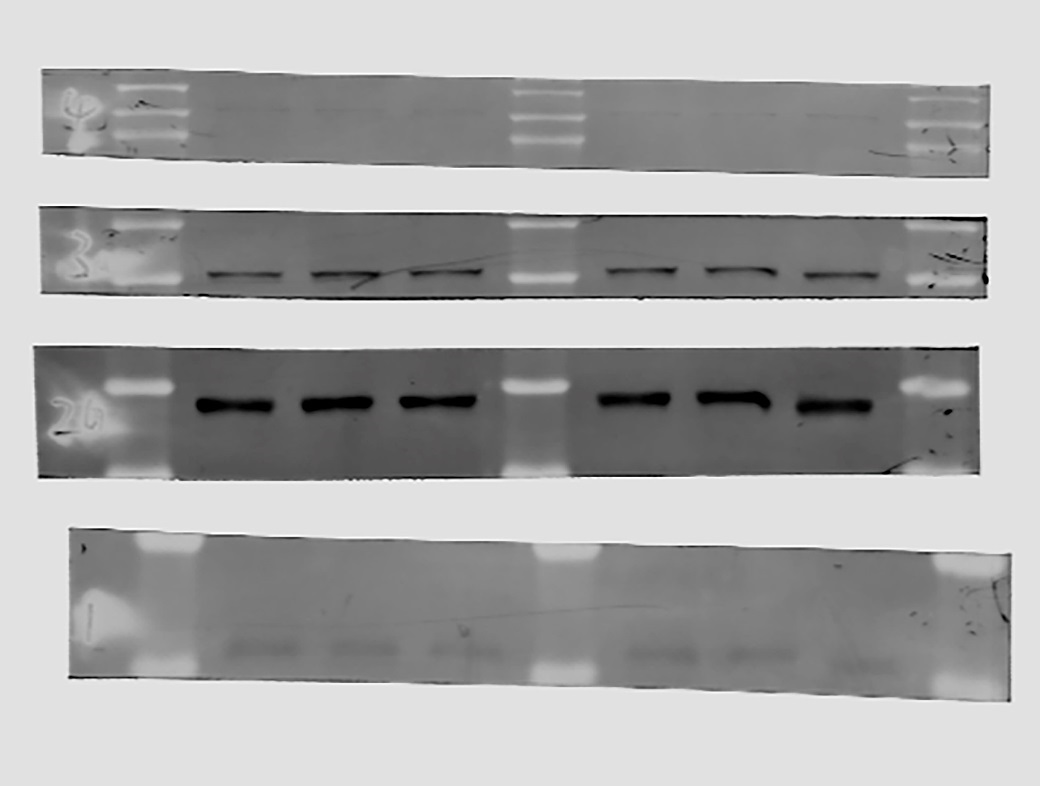


Note: Protein expression of GAPDH, SAAL1, Bcl-2 and Cyclin D1 in A549 cells.


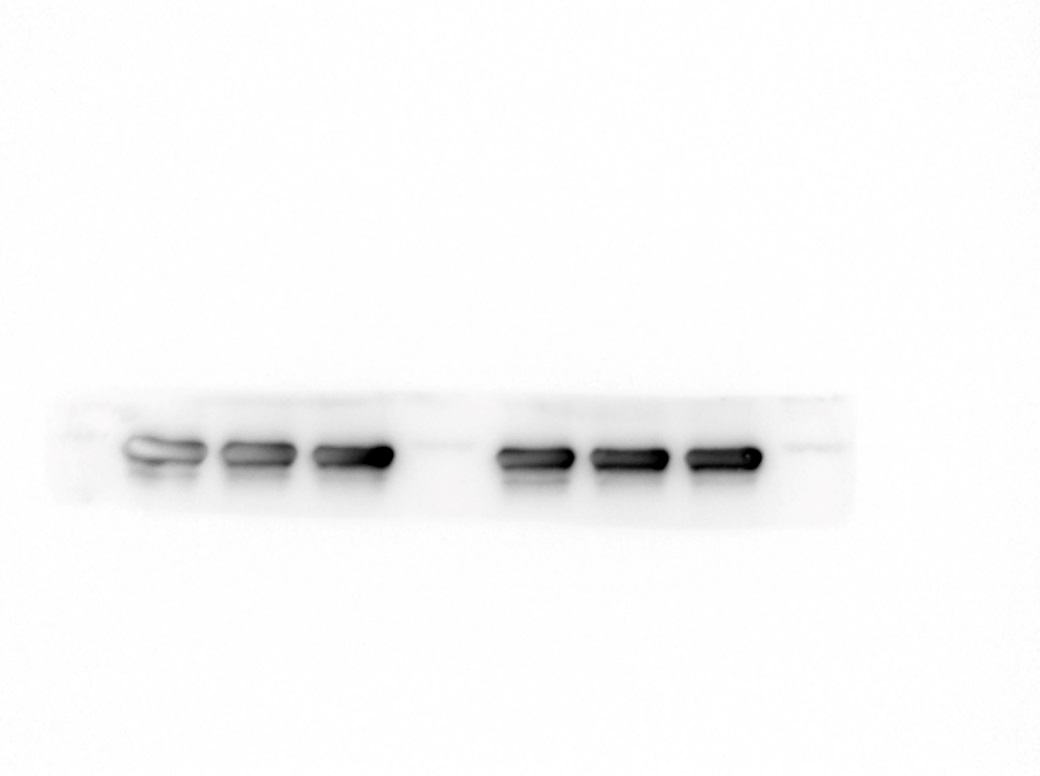


Note: Protein expression of GAPDH in A549 cells.


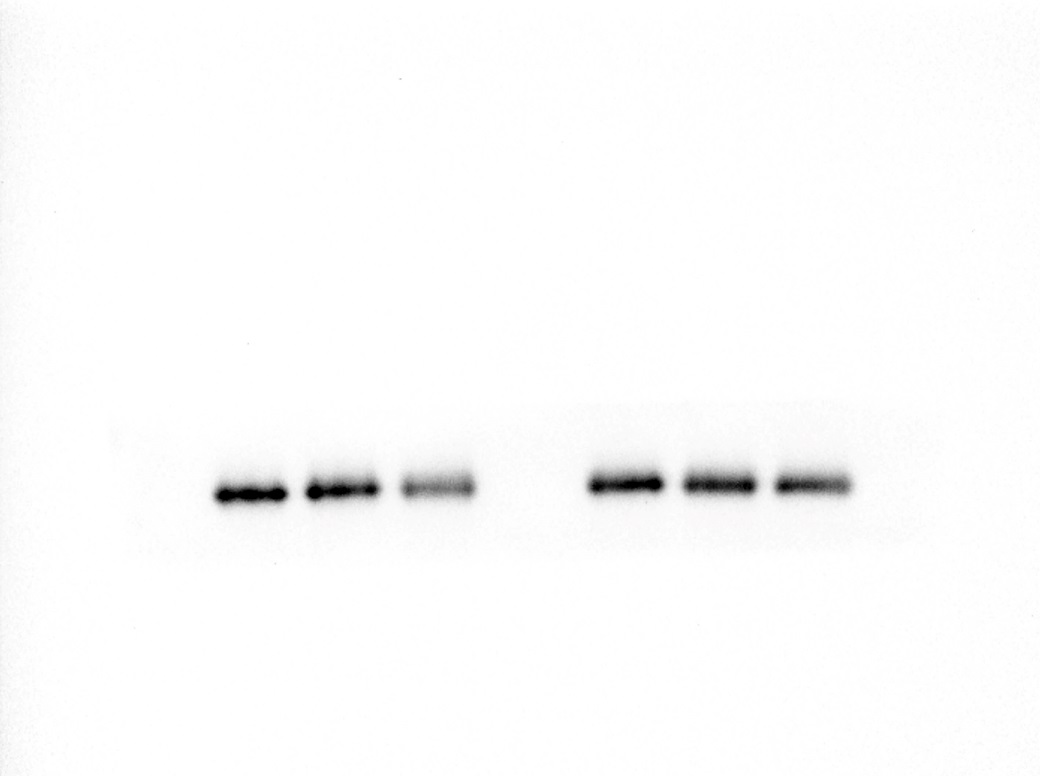


Note: Protein expression of SAAL1 in A549 cells.


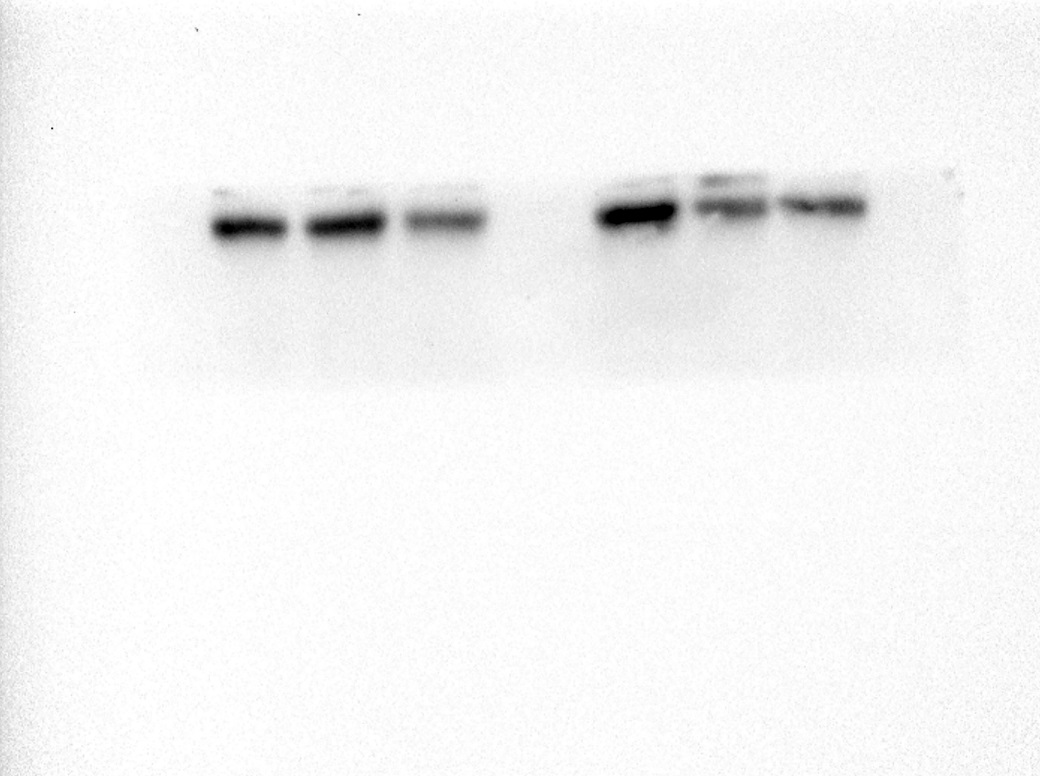


Note: Protein expression of Cyclin D1 in A549 cells.


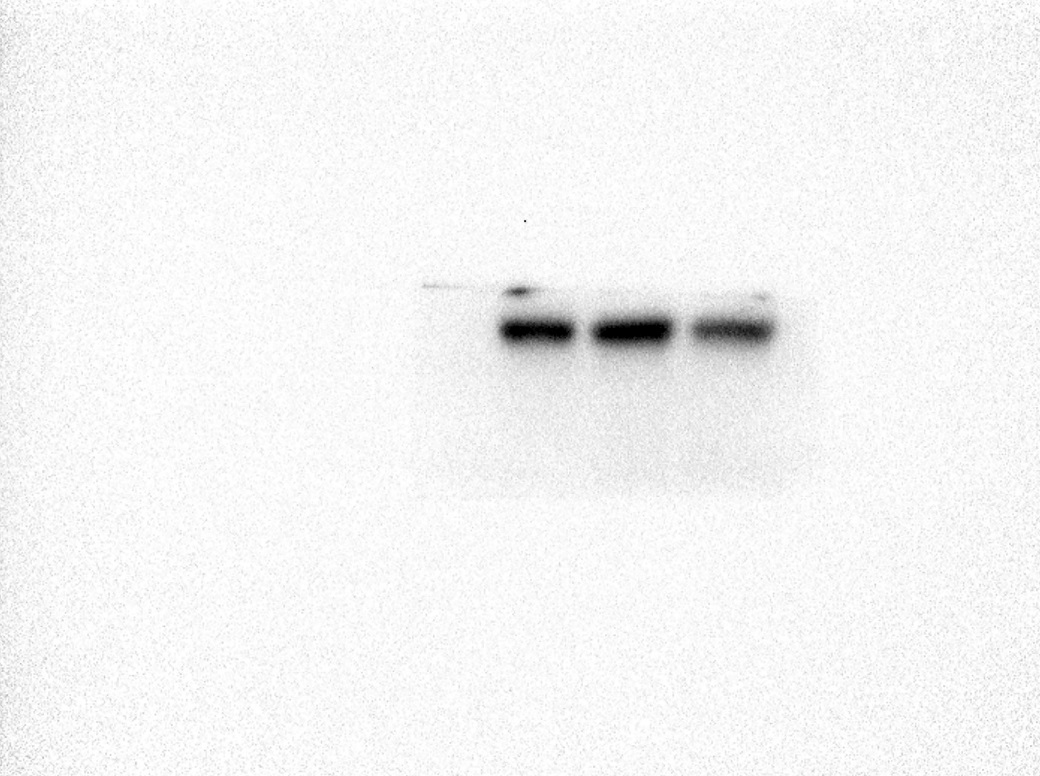


Note: Protein expression of Cyclin D1 in A549 cells.


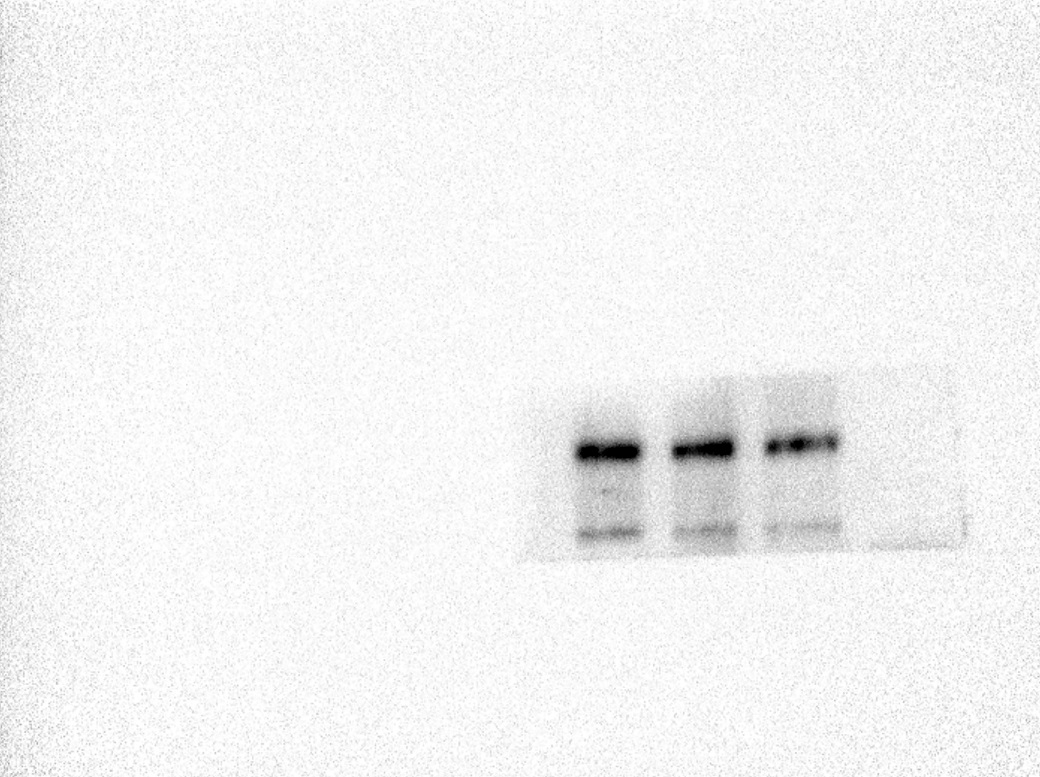


Note: Protein expression of Bcl-2 in A549 cells.

**Table S.**

Table S1. Functions of SAAL1-associated genes.

| Type | ID | Description | P |
| --- | --- | --- | --- |
| BP | GO:0007059 | chromosome segregation | 1.61E-26 |
| BP | GO:0000819 | sister chromatid segregation | 4.23E-25 |
| BP | GO:0140014 | mitotic nuclear division | 1.05E-23 |
| BP | GO:0000070 | mitotic sister chromatid segregation | 1.47E-23 |
| BP | GO:0000280 | nuclear division | 1.39E-22 |
| BP | GO:0098813 | nuclear chromosome segregation | 3.33E-22 |
| BP | GO:0048285 | organelle fission | 1.29E-21 |
| BP | GO:0006260 | DNA replication | 2.69E-20 |
| BP | GO:0006261 | DNA-dependent DNA replication | 4.89E-20 |
| BP | GO:0044786 | cell cycle DNA replication | 7.89E-18 |
| BP | GO:0033045 | regulation of sister chromatid segregation | 2.25E-15 |
| BP | GO:0051983 | regulation of chromosome segregation | 4.04E-14 |
| BP | GO:0045839 | negative regulation of mitotic nuclear division | 1.03E-13 |
| BP | GO:0071103 | DNA conformation change | 1.86E-13 |
| BP | GO:0010965 | regulation of mitotic sister chromatid separation | 2.48E-13 |
| BP | GO:0007088 | regulation of mitotic nuclear division | 2.55E-13 |
| BP | GO:0045930 | negative regulation of mitotic cell cycle | 3.01E-13 |
| BP | GO:0033260 | nuclear DNA replication | 4.04E-13 |
| BP | GO:0051306 | mitotic sister chromatid separation | 4.04E-13 |
| BP | GO:0051784 | negative regulation of nuclear division | 4.04E-13 |
| BP | GO:0051304 | chromosome separation | 4.20E-13 |
| BP | GO:1905818 | regulation of chromosome separation | 5.52E-13 |
| BP | GO:2000816 | negative regulation of mitotic sister chromatid separation | 6.01E-13 |
| BP | GO:1905819 | negative regulation of chromosome separation | 7.49E-13 |
| BP | GO:0033048 | negative regulation of mitotic sister chromatid segregation | 1.14E-12 |
| BP | GO:0051783 | regulation of nuclear division | 1.30E-12 |
| BP | GO:0033047 | regulation of mitotic sister chromatid segregation | 1.32E-12 |
| BP | GO:0033046 | negative regulation of sister chromatid segregation | 1.71E-12 |
| BP | GO:0051985 | negative regulation of chromosome segregation | 2.07E-12 |
| BP | GO:1901987 | regulation of cell cycle phase transition | 3.43E-12 |
| BP | GO:1901988 | negative regulation of cell cycle phase transition | 4.33E-12 |
| BP | GO:0030071 | regulation of mitotic metaphase/anaphase transition | 6.03E-12 |
| BP | GO:1902099 | regulation of metaphase/anaphase transition of cell cycle | 8.34E-12 |
| BP | GO:0007091 | metaphase/anaphase transition of mitotic cell cycle | 9.77E-12 |
| BP | GO:0010948 | negative regulation of cell cycle process | 1.26E-11 |
| BP | GO:0044784 | metaphase/anaphase transition of cell cycle | 1.33E-11 |
| BP | GO:1901990 | regulation of mitotic cell cycle phase transition | 1.50E-11 |
| BP | GO:0045841 | negative regulation of mitotic metaphase/anaphase transition | 2.73E-11 |
| BP | GO:1903046 | meiotic cell cycle process | 3.14E-11 |
| BP | GO:1902100 | negative regulation of metaphase/anaphase transition of cell cycle | 3.36E-11 |
| BP | GO:1901991 | negative regulation of mitotic cell cycle phase transition | 3.39E-11 |
| BP | GO:0007062 | sister chromatid cohesion | 3.55E-11 |
| BP | GO:0006271 | DNA strand elongation involved in DNA replication | 4.32E-11 |
| BP | GO:0000082 | G1/S transition of mitotic cell cycle | 1.33E-10 |
| BP | GO:0000075 | cell cycle checkpoint | 1.40E-10 |
| BP | GO:0007093 | mitotic cell cycle checkpoint | 1.89E-10 |
| BP | GO:0044843 | cell cycle G1/S phase transition | 2.83E-10 |
| BP | GO:0140013 | meiotic nuclear division | 2.84E-10 |
| BP | GO:0022616 | DNA strand elongation | 3.24E-10 |
| BP | GO:0032201 | telomere maintenance via semi-conservative replication | 3.24E-10 |
| BP | GO:0051321 | meiotic cell cycle | 6.36E-10 |
| BP | GO:0033044 | regulation of chromosome organization | 1.36E-09 |
| BP | GO:0007094 | mitotic spindle assembly checkpoint | 1.44E-09 |
| BP | GO:0031577 | spindle checkpoint | 1.44E-09 |
| BP | GO:0071173 | spindle assembly checkpoint | 1.44E-09 |
| BP | GO:0071174 | mitotic spindle checkpoint | 1.44E-09 |
| BP | GO:0032508 | DNA duplex unwinding | 3.32E-09 |
| BP | GO:0051303 | establishment of chromosome localization | 5.86E-09 |
| BP | GO:0032392 | DNA geometric change | 6.21E-09 |
| BP | GO:0050000 | chromosome localization | 6.44E-09 |
| BP | GO:0007080 | mitotic metaphase plate congression | 7.39E-09 |
| BP | GO:0000724 | double-strand break repair via homologous recombination | 1.59E-08 |
| BP | GO:0000725 | recombinational repair | 1.68E-08 |
| BP | GO:0045132 | meiotic chromosome segregation | 2.12E-08 |
| BP | GO:0090329 | regulation of DNA-dependent DNA replication | 2.63E-08 |
| BP | GO:2001251 | negative regulation of chromosome organization | 3.11E-08 |
| BP | GO:0051310 | metaphase plate congression | 3.67E-08 |
| BP | GO:0090068 | positive regulation of cell cycle process | 5.59E-08 |
| BP | GO:0070601 | centromeric sister chromatid cohesion | 5.71E-08 |
| BP | GO:0045787 | positive regulation of cell cycle | 6.51E-08 |
| BP | GO:0006302 | double-strand break repair | 1.41E-07 |
| BP | GO:0006270 | DNA replication initiation | 1.49E-07 |
| BP | GO:0072401 | signal transduction involved in DNA integrity checkpoint | 1.65E-07 |
| BP | GO:0072422 | signal transduction involved in DNA damage checkpoint | 1.65E-07 |
| BP | GO:0072395 | signal transduction involved in cell cycle checkpoint | 1.79E-07 |
| BP | GO:0044839 | cell cycle G2/M phase transition | 2.55E-07 |
| BP | GO:0072331 | signal transduction by p53 class mediator | 2.64E-07 |
| BP | GO:0034501 | protein localization to kinetochore | 2.71E-07 |
| BP | GO:1902850 | microtubule cytoskeleton organization involved in mitosis | 2.84E-07 |
| BP | GO:0034502 | protein localization to chromosome | 3.32E-07 |
| BP | GO:0006310 | DNA recombination | 5.59E-07 |
| BP | GO:0071459 | protein localization to chromosome, centromeric region | 6.75E-07 |
| BP | GO:0010639 | negative regulation of organelle organization | 7.55E-07 |
| BP | GO:0007064 | mitotic sister chromatid cohesion | 9.93E-07 |
| BP | GO:0006977 | DNA damage response, signal transduction by p53  class mediator resulting in cell cycle arrest | 1.25E-06 |
| BP | GO:0072431 | signal transduction involved in mitotic G1 DNA  damage checkpoint | 1.36E-06 |
| BP | GO:1902400 | intracellular signal transduction involved in G1  DNA damage checkpoint | 1.36E-06 |
| BP | GO:0007052 | mitotic spindle organization | 1.52E-06 |
| BP | GO:0072413 | signal transduction involved in mitotic cell cycle checkpoint | 1.62E-06 |
| BP | GO:1902402 | signal transduction involved in mitotic DNA damage checkpoint | 1.62E-06 |
| BP | GO:1902403 | signal transduction involved in mitotic DNA integrity checkpoint | 1.62E-06 |
| BP | GO:0007051 | spindle organization | 1.65E-06 |
| BP | GO:0006275 | regulation of DNA replication | 1.69E-06 |
| BP | GO:0031571 | mitotic G1 DNA damage checkpoint | 2.25E-06 |
| BP | GO:0044819 | mitotic G1/S transition checkpoint | 2.25E-06 |
| BP | GO:0044783 | G1 DNA damage checkpoint | 2.43E-06 |
| BP | GO:0007135 | meiosis II | 4.32E-06 |
| BP | GO:0032875 | regulation of DNA endoreduplication | 4.32E-06 |
| BP | GO:0061983 | meiosis II cell cycle process | 4.32E-06 |
| BP | GO:0042770 | signal transduction in response to DNA damage | 5.68E-06 |
| BP | GO:0000727 | double-strand break repair via break-induced replication | 5.93E-06 |
| BP | GO:0042023 | DNA endoreduplication | 5.93E-06 |
| BP | GO:0006323 | DNA packaging | 6.69E-06 |
| BP | GO:1902749 | regulation of cell cycle G2/M phase transition | 7.34E-06 |
| BP | GO:0031145 | anaphase-promoting complex-dependent catabolic process | 7.83E-06 |
| BP | GO:0007077 | mitotic nuclear envelope disassembly | 7.88E-06 |
| BP | GO:0016572 | histone phosphorylation | 7.97E-06 |
| BP | GO:0071158 | positive regulation of cell cycle arrest | 8.32E-06 |
| BP | GO:0000077 | DNA damage checkpoint | 9.34E-06 |
| BP | GO:0051052 | regulation of DNA metabolic process | 1.30E-05 |
| BP | GO:1901992 | positive regulation of mitotic cell cycle phase transition | 1.38E-05 |
| BP | GO:0007050 | cell cycle arrest | 1.47E-05 |
| BP | GO:0031570 | DNA integrity checkpoint | 1.47E-05 |
| BP | GO:1902969 | mitotic DNA replication | 1.62E-05 |
| BP | GO:0000723 | telomere maintenance | 1.76E-05 |
| BP | GO:0045931 | positive regulation of mitotic cell cycle | 1.82E-05 |
| BP | GO:0030261 | chromosome condensation | 1.88E-05 |
| BP | GO:0044773 | mitotic DNA damage checkpoint | 1.89E-05 |
| BP | GO:0000086 | G2/M transition of mitotic cell cycle | 1.92E-05 |
| BP | GO:0030397 | membrane disassembly | 1.99E-05 |
| BP | GO:0051081 | nuclear envelope disassembly | 1.99E-05 |
| BP | GO:0007076 | mitotic chromosome condensation | 2.41E-05 |
| BP | GO:0032200 | telomere organization | 2.72E-05 |
| BP | GO:0044774 | mitotic DNA integrity checkpoint | 2.90E-05 |
| BP | GO:1901989 | positive regulation of cell cycle phase transition | 2.90E-05 |
| BP | GO:0030330 | DNA damage response, signal transduction by p53 class mediator | 3.04E-05 |
| BP | GO:0036297 | interstrand cross-link repair | 3.04E-05 |
| BP | GO:0071156 | regulation of cell cycle arrest | 3.18E-05 |
| BP | GO:0042276 | error-prone translesion synthesis | 4.01E-05 |
| BP | GO:0051383 | kinetochore organization | 4.66E-05 |
| BP | GO:0007063 | regulation of sister chromatid cohesion | 5.39E-05 |
| BP | GO:2000134 | negative regulation of G1/S transition of mitotic cell cycle | 6.40E-05 |
| BP | GO:0070192 | chromosome organization involved in meiotic cell cycle | 6.83E-05 |
| BP | GO:1902807 | negative regulation of cell cycle G1/S phase transition | 7.99E-05 |
| BP | GO:0007292 | female gamete generation | 9.54E-05 |
| BP | GO:0010971 | positive regulation of G2/M transition of mitotic cell cycle | 0.000101089 |
| BP | GO:0051984 | positive regulation of chromosome segregation | 0.000112947 |
| BP | GO:1902751 | positive regulation of cell cycle G2/M phase transition | 0.000139305 |
| BP | GO:0007143 | female meiotic nuclear division | 0.00015386 |
| BP | GO:0008608 | attachment of spindle microtubules to kinetochore | 0.000169369 |
| BP | GO:0019985 | translesion synthesis | 0.000356215 |
| BP | GO:2000045 | regulation of G1/S transition of mitotic cell cycle | 0.000389659 |
| BP | GO:0070507 | regulation of microtubule cytoskeleton organization | 0.00040943 |
| BP | GO:0034080 | CENP-A containing nucleosome assembly | 0.000439385 |
| BP | GO:0061641 | CENP-A containing chromatin organization | 0.000439385 |
| BP | GO:0010569 | regulation of double-strand break repair via  homologous recombination | 0.00046964 |
| BP | GO:0010389 | regulation of G2/M transition of mitotic cell cycle | 0.000519893 |
| BP | GO:0071897 | DNA biosynthetic process | 0.000519893 |
| BP | GO:0031109 | microtubule polymerization or depolymerization | 0.000556642 |
| BP | GO:0031055 | chromatin remodeling at centromere | 0.000568329 |
| BP | GO:1902806 | regulation of cell cycle G1/S phase transition | 0.000596138 |
| BP | GO:0051445 | regulation of meiotic cell cycle | 0.000603943 |
| BP | GO:0035404 | histone-serine phosphorylation | 0.000604385 |
| BP | GO:0045835 | negative regulation of meiotic nuclear division | 0.000604385 |
| BP | GO:0000731 | DNA synthesis involved in DNA repair | 0.000679379 |
| BP | GO:0061982 | meiosis I cell cycle process | 0.000699275 |
| BP | GO:0006301 | postreplication repair | 0.000719246 |
| BP | GO:0006998 | nuclear envelope organization | 0.000719246 |
| BP | GO:0060707 | trophoblast giant cell differentiation | 0.000723685 |
| BP | GO:2000105 | positive regulation of DNA-dependent DNA replication | 0.000723685 |
| BP | GO:2001020 | regulation of response to DNA damage stimulus | 0.000773502 |
| BP | GO:0006336 | DNA replication-independent nucleosome assembly | 0.000803384 |
| BP | GO:0032886 | regulation of microtubule-based process | 0.000840583 |
| BP | GO:0034724 | DNA replication-independent nucleosome organization | 0.000847697 |
| BP | GO:0018107 | peptidyl-threonine phosphorylation | 0.000866054 |
| BP | GO:0034508 | centromere complex assembly | 0.000893534 |
| BP | GO:0006268 | DNA unwinding involved in DNA replication | 0.000993476 |
| BP | GO:0090231 | regulation of spindle checkpoint | 0.000993476 |
| BP | GO:0090266 | regulation of mitotic cell cycle spindle assembly checkpoint | 0.000993476 |
| BP | GO:1903504 | regulation of mitotic spindle checkpoint | 0.000993476 |
| BP | GO:0051054 | positive regulation of DNA metabolic process | 0.001027262 |
| BP | GO:0018210 | peptidyl-threonine modification | 0.001088969 |
| BP | GO:0043486 | histone exchange | 0.001092529 |
| BP | GO:0070365 | hepatocyte differentiation | 0.001143829 |
| BP | GO:0022412 | cellular process involved in reproduction in  multicellular organism | 0.001272354 |
| BP | GO:0051382 | kinetochore assembly | 0.001304394 |
| BP | GO:0051447 | negative regulation of meiotic cell cycle | 0.001304394 |
| BP | GO:0065004 | protein-DNA complex assembly | 0.001491014 |
| BP | GO:2000573 | positive regulation of DNA biosynthetic process | 0.001504008 |
| BP | GO:0051782 | negative regulation of cell division | 0.001655889 |
| BP | GO:0070987 | error-free translesion synthesis | 0.002047418 |
| BP | GO:2000779 | regulation of double-strand break repair | 0.002242277 |
| BP | GO:2001021 | negative regulation of response to DNA damage stimulus | 0.00259043 |
| BP | GO:0007018 | microtubule-based movement | 0.002650314 |
| BP | GO:0000910 | cytokinesis | 0.002658117 |
| BP | GO:0006297 | nucleotide-excision repair, DNA gap filling | 0.002708599 |
| BP | GO:0071824 | protein-DNA complex subunit organization | 0.002857744 |
| BP | GO:1901796 | regulation of signal transduction by p53 class mediator | 0.00319647 |
| BP | GO:0051307 | meiotic chromosome separation | 0.00319787 |
| BP | GO:0043044 | ATP-dependent chromatin remodeling | 0.003276468 |
| BP | GO:0006338 | chromatin remodeling | 0.003325482 |
| BP | GO:0032465 | regulation of cytokinesis | 0.003382675 |
| BP | GO:0001556 | oocyte maturation | 0.003456853 |
| BP | GO:0060706 | cell differentiation involved in embryonic placenta development | 0.003456853 |
| BP | GO:0060249 | anatomical structure homeostasis | 0.003502229 |
| BP | GO:0071168 | protein localization to chromatin | 0.004003189 |
| BP | GO:0000083 | regulation of transcription involved in G1/S  transition of mitotic cell cycle | 0.004290412 |
| BP | GO:0000132 | establishment of mitotic spindle orientation | 0.004290412 |
| BP | GO:0000018 | regulation of DNA recombination | 0.004823796 |
| BP | GO:0040020 | regulation of meiotic nuclear division | 0.004892647 |
| BP | GO:0000079 | regulation of cyclin-dependent protein serine/  threonine kinase activity | 0.004958136 |
| BP | GO:1901976 | regulation of cell cycle checkpoint | 0.005207533 |
| BP | GO:1904029 | regulation of cyclin-dependent protein kinase activity | 0.005518042 |
| BP | GO:0040001 | establishment of mitotic spindle localization | 0.005531512 |
| BP | GO:0051225 | spindle assembly | 0.005811664 |
| BP | GO:2000278 | regulation of DNA biosynthetic process | 0.005811664 |
| BP | GO:0051294 | establishment of spindle orientation | 0.0062065 |
| BP | GO:0045740 | positive regulation of DNA replication | 0.006557383 |
| BP | GO:0007127 | meiosis I | 0.006748131 |
| BP | GO:0006296 | nucleotide-excision repair, DNA incision, 5'-to lesion | 0.006917111 |
| BP | GO:0033683 | nucleotide-excision repair, DNA incision | 0.007662851 |
| BP | GO:0042769 | DNA damage response, detection of DNA damage | 0.007662851 |
| BP | GO:0006282 | regulation of DNA repair | 0.008129113 |
| BP | GO:0001701 | in utero embryonic development | 0.008443516 |
| BP | GO:0051293 | establishment of spindle localization | 0.00925776 |
| BP | GO:0006997 | nucleus organization | 0.009665029 |
| BP | GO:0007019 | microtubule depolymerization | 0.009677683 |
| BP | GO:0048599 | oocyte development | 0.010542544 |
| BP | GO:0051653 | spindle localization | 0.011440366 |
| BP | GO:0009994 | oocyte differentiation | 0.012847875 |
| BP | GO:0006334 | nucleosome assembly | 0.012973876 |
| BP | GO:2000241 | regulation of reproductive process | 0.013214698 |
| BP | GO:0043161 | proteasome-mediated ubiquitin-dependent  protein catabolic process | 0.013469014 |
| BP | GO:0045840 | positive regulation of mitotic nuclear division | 0.013826058 |
| BP | GO:2000242 | negative regulation of reproductive process | 0.01483558 |
| BP | GO:0048608 | reproductive structure development | 0.015054226 |
| BP | GO:0090307 | mitotic spindle assembly | 0.015351952 |
| BP | GO:0061458 | reproductive system development | 0.015469318 |
| BP | GO:0009314 | response to radiation | 0.017508251 |
| BP | GO:0031497 | chromatin assembly | 0.018279282 |
| BP | GO:0090305 | nucleic acid phosphodiester bond hydrolysis | 0.018950393 |
| BP | GO:0051302 | regulation of cell division | 0.019164549 |
| BP | GO:0051785 | positive regulation of nuclear division | 0.020928152 |
| BP | GO:2001252 | positive regulation of chromosome organization | 0.021005574 |
| BP | GO:0002478 | antigen processing and presentation of exogenous peptide antigen | 0.02132156 |
| BP | GO:0010498 | proteasomal protein catabolic process | 0.022284421 |
| BP | GO:0019884 | antigen processing and presentation of exogenous antigen | 0.023606739 |
| BP | GO:0034728 | nucleosome organization | 0.024283205 |
| BP | GO:0000281 | mitotic cytokinesis | 0.025258361 |
| BP | GO:0006283 | transcription-coupled nucleotide-excision repair | 0.025258361 |
| BP | GO:0048002 | antigen processing and presentation of peptide antigen | 0.026020158 |
| BP | GO:0006333 | chromatin assembly or disassembly | 0.026733244 |
| BP | GO:0090502 | RNA phosphodiester bond hydrolysis, endonucleolytic | 0.027882476 |
| BP | GO:0031110 | regulation of microtubule polymerization or depolymerization | 0.029234032 |
| BP | GO:0007084 | mitotic nuclear envelope reassembly | 0.033244055 |
| BP | GO:0034085 | establishment of sister chromatid cohesion | 0.033244055 |
| BP | GO:0045876 | positive regulation of sister chromatid cohesion | 0.033244055 |
| BP | GO:0071281 | cellular response to iron ion | 0.033244055 |
| BP | GO:1902101 | positive regulation of metaphase/anaphase transition of cell cycle | 0.033244055 |
| BP | GO:1903862 | positive regulation of oxidative phosphorylation | 0.033244055 |
| CC | GO:0098687 | chromosomal region | 4.02E-23 |
| CC | GO:0000775 | chromosome, centromeric region | 5.36E-22 |
| CC | GO:0000776 | kinetochore | 1.42E-19 |
| CC | GO:0000793 | condensed chromosome | 2.80E-19 |
| CC | GO:0000779 | condensed chromosome, centromeric region | 9.99E-19 |
| CC | GO:0000777 | condensed chromosome kinetochore | 1.08E-17 |
| CC | GO:0005819 | spindle | 1.04E-14 |
| CC | GO:0000940 | condensed chromosome outer kinetochore | 2.45E-12 |
| CC | GO:0000780 | condensed nuclear chromosome, centromeric region | 1.83E-10 |
| CC | GO:0000778 | condensed nuclear chromosome kinetochore | 8.30E-10 |
| CC | GO:0000794 | condensed nuclear chromosome | 9.35E-10 |
| CC | GO:0005657 | replication fork | 9.32E-08 |
| CC | GO:0005874 | microtubule | 6.56E-06 |
| CC | GO:0042555 | MCM complex | 6.70E-06 |
| CC | GO:0043596 | nuclear replication fork | 8.77E-06 |
| CC | GO:0000922 | spindle pole | 1.39E-05 |
| CC | GO:0005871 | kinesin complex | 2.86E-05 |
| CC | GO:0005881 | cytoplasmic microtubule | 7.00E-05 |
| CC | GO:0072686 | mitotic spindle | 0.000409579 |
| CC | GO:0008278 | cohesin complex | 0.001171433 |
| CC | GO:0005875 | microtubule associated complex | 0.001420625 |
| CC | GO:0061695 | transferase complex, transferring  phosphorus-containing groups | 0.001421608 |
| CC | GO:0000781 | chromosome, telomeric region | 0.001754291 |
| CC | GO:0043601 | nuclear replisome | 0.002650324 |
| CC | GO:0030894 | replisome | 0.003108055 |
| CC | GO:0032993 | protein-DNA complex | 0.003974951 |
| CC | GO:0000784 | nuclear chromosome, telomeric region | 0.007489657 |
| CC | GO:0000307 | cyclin-dependent protein kinase holoenzyme complex | 0.007968217 |
| CC | GO:0005876 | spindle microtubule | 0.015294214 |
| CC | GO:0045171 | intercellular bridge | 0.015294214 |
| CC | GO:1902554 | serine/threonine protein kinase complex | 0.032259695 |
| CC | GO:0000235 | astral microtubule | 0.034599802 |
| CC | GO:0005652 | nuclear lamina | 0.034599802 |
| CC | GO:0005818 | aster | 0.034599802 |
| CC | GO:0072687 | meiotic spindle | 0.034599802 |
| MF | GO:0140097 | catalytic activity, acting on DNA | 1.46E-10 |
| MF | GO:0003678 | DNA helicase activity | 3.27E-10 |
| MF | GO:0004386 | helicase activity | 8.48E-08 |
| MF | GO:0035173 | histone kinase activity | 2.94E-07 |
| MF | GO:0017116 | single-stranded DNA-dependent ATP-dependent  DNA helicase activity | 5.94E-07 |
| MF | GO:0043142 | single-stranded DNA-dependent ATPase activity | 5.94E-07 |
| MF | GO:0004003 | ATP-dependent DNA helicase activity | 7.32E-07 |
| MF | GO:0008026 | ATP-dependent helicase activity | 7.32E-07 |
| MF | GO:0070035 | purine NTP-dependent helicase activity | 7.32E-07 |
| MF | GO:0043138 | 3'-5' DNA helicase activity | 4.26E-05 |
| MF | GO:0003697 | single-stranded DNA binding | 4.34E-05 |
| MF | GO:0016887 | ATPase activity | 4.93E-05 |
| MF | GO:0003688 | DNA replication origin binding | 7.49E-05 |
| MF | GO:0008094 | DNA-dependent ATPase activity | 9.34E-05 |
| MF | GO:0003777 | microtubule motor activity | 0.000200594 |
| MF | GO:0004523 | RNA-DNA hybrid ribonuclease activity | 0.000630056 |
| MF | GO:0004674 | protein serine/threonine kinase activity | 0.000757114 |
| MF | GO:0003774 | motor activity | 0.001240542 |
| MF | GO:0008017 | microtubule binding | 0.001572983 |
| MF | GO:0042623 | ATPase activity, coupled | 0.002936522 |
| MF | GO:0003887 | DNA-directed DNA polymerase activity | 0.0038808 |
| MF | GO:0015631 | tubulin binding | 0.005974975 |
| MF | GO:0016891 | endoribonuclease activity, producing 5'-phosphomonoesters | 0.00610741 |
| MF | GO:0034061 | DNA polymerase activity | 0.007586033 |
| MF | GO:0016893 | endonuclease activity, active with either ribo- or  deoxyribonucleic acids and producing 5'-phosphomonoesters | 0.010973406 |
| MF | GO:0016779 | nucleotidyltransferase activity | 0.011778517 |
| MF | GO:0016538 | cyclin-dependent protein serine/threonine kinase regulator activity | 0.012386266 |
| MF | GO:0004521 | endoribonuclease activity | 0.020545449 |
| MF | GO:0003684 | damaged DNA binding | 0.021151272 |
